# Hypergravity exposure leads to persistent effects on geotaxis and activity in *Drosophila melanogaster*

**DOI:** 10.1242/jeb.251327

**Published:** 2026-04-23

**Authors:** Sushmita Arumugam Amogh, Savannah Horton, Ysabel Milton Giraldo

**Affiliations:** ^1^ Neuroscience Graduate Program, University of California, Riverside, Riverside, CA 92521, USA; ^2^ Department of Entomology, University of California, Riverside, Riverside, CA 92521, USA

**Keywords:** Locomotion, Gravity, Hyperactivity, Climbing, Development, Insect, Fly, Long-term effects

## Abstract

Gravity, a constant force throughout evolution, has fundamentally shaped biology, playing a critical role in locomotion, balance and orientation across species – from unicellular organisms to complex multicellular life. Despite its pivotal role in biomechanics and physiology, how gravity affects different aspects of locomotion remains less understood. Hypergravity induces changes in activity rhythms across taxa. However, comprehensive analyses testing varied gravity intensities and examining how acute or chronic developmental exposure affects locomotion after returning to Earth's gravity remain limited. Using a hypergravity simulator, we found that acute 4 ***g*** exposure impairs spontaneous climbing behavior in *Drosophila melanogaster*, while startle-induced climbing remains unaffected when tested at 1 ***g***. After exposure to higher gravity levels (7 ***g***, 10 ***g*** and 13 ***g***), spontaneous climbing deficits become more pronounced, yet startle responses remain largely intact. When examining individual daily activity over 1 week post-exposure, we observed increased activity following 4 ***g*** exposure but reduced activity after exposure to higher gravity levels. Notably, these locomotor impairments persisted beyond the exposure period, with recovery occurring later in life, indicating long-lasting effects of hypergravity. In parallel, whole-body triacylglyceride measurements revealed gravity- and time-dependent modulation of energy storage following hypergravity exposure. To explore the effects of developmental and multigenerational exposure, we subjected flies to hypergravity for one or ten generations. Chronic treatment in both cases further reduced activity relative to acute exposure, even at 4 ***g***, with more pronounced reductions at 7 ***g***. Together, our findings demonstrate that altered gravity exposure modulates locomotion, with lasting consequences for activity and energy homeostasis after return to 1 ***g***.

## Teaser

**Summary:** Gravity changes affect fly behavior differently depending on movement type, hypergravity intensity and duration of exposure, revealing how adult or developmental exposure to hypergravity alters later locomotion and energy use at Earth's gravity.

## INTRODUCTION

Gravity is a fundamental force that governs movement across all biological scales (Alpatov et al., 1994; Fuller, 1984; Hoban-Higgins et al., 1995; Hosamani et al., 2022; Sineshchekov et al., 2000; Tays et al., 2021). From single-celled organisms to complex vertebrates, locomotor strategies have evolved to operate under Earth's constant gravitational force of 1 ***g***. At the microscopic level, many unicellular organisms exhibit gravitaxis, actively orienting their movement relative to gravity (Häder et al., 2005). In multicellular organisms, gravity shapes posture and movement efficiency. Many aquatic animals experience a partial offset of gravity's effects due to buoyancy, which reduces effective body weight and allows for more energy-efficient movement in swimming species (Alexander, 1982; Anderson and Grosenbaugh, 2005; Jobling, 2010; Vogel, 1996). As animals evolved from water to land and, and in some lineages, to air, terrestrial taxa adapted musculoskeletal and vestibular systems to resist gravitational pull (Angelaki and Cullen, 2008; Biewener, 1989; Lacquaniti et al., 2014; Massion, 1994; Proske and Gandevia, 2012), and flying animals use wing motion and lift to stay aloft (Dudley, 2000; Norberg, 1990; Sane, 2003; Tobalske, 2007). In humans, finely tuned gait dynamics, balance and proprioception enable stable, energy-efficient movement under gravity's influence (Alexander, 1991; Horak, 2006; Schmitt, 2003; Winter, 1995). Many amphibious and semi-aquatic animals, such as frogs (Emerson, 1978; Nauwelaerts et al., 2005), mudskippers (Bauer et al., 2020; Pace and Gibb, 2011) and seals (Fish, 1999; Garrett and Fish, 2015), naturally transition between water and land, repeatedly adjusting to changes in buoyancy and weight support to move efficiently. During human space missions, astronauts transition between Earth's gravity and microgravity and must re-adapt their locomotor and vestibular systems upon returning to Earth (Clément et al., 2020; Wuyts et al., 2025).

Experimentally altering Earth's constant gravitational force – by decreasing it (microgravity, <1 ***g***) or increasing it (hypergravity, >1 ***g***) – provides a means to uncover how gravity shapes movement. Hypergravity is a practical way to increase weight bearing on Earth and helps test gravity's role alongside microgravity studies (Clément et al., 2015; van Loon, 2016). Thus, researchers can experimentally use controlled hypergravity to reveal how varying loads and transitions back to 1 ***g*** uncover core physiological mechanisms of locomotor adjustment. Microgravity studies have consistently shown that astronauts, rodents and insects exhibit impaired balance, altered locomotor coordination, gait instability and sensorimotor dysfunction upon returning to Earth's gravity (Bloomberg et al., 1997; Clément et al., 2020; Lackner and DiZio, 2000; Mhatre et al., 2022; Paloski et al., 1993; Reschke et al., 1998; Walton et al., 2005; Wood et al., 2015). Exposure to hypergravity is known to be energetically demanding, requiring increased muscular effort for movement, as shown in studies assessing physiological and metabolic changes during exposure (Abe et al., 2022; Economos et al., 1982; Lints and Le Bourg, 1989; Minois, 2006; Richoilley et al., 1988; Thorling and Fredens, 1995), reflected in elevated glucose utilization, increased metabolic rates and greater muscular activity. Additionally, studies in rodents report persistent effects of exposure post-hypergravity, including altered glucose metabolism, increased bone density and muscle weight, and long-lasting neurophysiological adaptations such as changes in vestibular reflexes (Bojados and Jamon, 2014; Daligcon and Oyama, 1975; Tominari et al., 2019). Exposure to hypergravity has direct effects on the musculoskeletal system, increasing muscle load, altering muscle function, improving neuromuscular performance and increasing muscle and bone mass, both during exposure and after returning to 1 ***g*** (Bojados and Jamon, 2014; Bosco, 1985; Bosco et al., 1984; Schilder and Raynor, 2017; Tominari et al., 2019). While studies in rodents have shown that developmental or adult hypergravity exposure can lead to persistent locomotor impairments – such as altered gait patterns, postural control and motor coordination upon return to 1 ***g*** (Bojados et al., 2013; Bouët et al., 2004; Nguon et al., 2006; Noh et al., 2020) – such post-exposure effects remain less well characterized in invertebrate models such as *Drosophila*. With their short generation time and ease of rearing, *Drosophila* provide a unique opportunity to investigate how hypergravity influences behavior and physiology across multiple generations – an approach that is difficult to achieve in vertebrate systems. Our study addresses this gap by examining how hypergravity intensity and duration affect climbing, walking and daily activity following return to Earth's gravity throughout the flies' lifespan and across generations.

Organisms commonly show distinct physiological and behavioral responses to short-term versus prolonged environmental stressors – a well-established principle in comparative physiology and ecological adaptation (Kültz, 2005; Seebacher and Franklin, 2012). Chronic exposure throughout development to environmental stressors such as nutritional restriction, temperature stress or hypoxia is known to induce structural and functional plasticity, including changes in neuromuscular junction growth, synaptic function and locomotor behavior (Grandison et al., 2009; Robinson et al., 2012; Wingrove and O'Farrell, 1999; Zhong and Wu, 2004). In the present study, we used climbing and daily activity monitoring assays to examine how flies respond to both acute and chronic hypergravity exposure. This framework allowed us to compare short-term compensatory behavioral or physiological responses with long-term adaptations that may arise through developmental plasticity under sustained hypergravity.

In *Drosophila melanogaster*, acute hypergravity exposure has been shown to affect locomotion across several contexts. Earlier studies reported changes in negative geotaxis and climbing after short-term exposure to elevated ***g***-levels (Le Bourg and Minois, 1999), and more recent work has demonstrated impaired startle-induced climbing and muscle plasticity at 12 ***g*** (Schilder and Raynor, 2017). Experiments at 2 ***g*** have further revealed alterations in walking behavior, motility and survival, with strain-specific differences in sensitivity (Hill et al., 2012; Serrano et al., 2013). Chronic hypergravity exposure has also been examined, including long-term adult exposure (70 days) affecting lifespan and fecundity (Lints and Le Bourg, 1989). Together, these studies show that hypergravity can influence behavioral performance, but they have generally focused on one or a few ***g***-levels, specific assay types or a single exposure duration. As a result, we lack an understanding of how different locomotor behaviors respond across a broader range of gravity levels and exposure durations, and whether hypergravity-induced changes persist after flies return to 1 ***g***. Additionally, while hypergravity may influence energetic demands, to our knowledge, its effects on whole-body lipid stores have not been examined. We hypothesized that increasing hypergravity levels would progressively impair climbing, walking and activity upon return to 1 ***g***, reflecting physiological adjustment to altered gravitational loads. We also predicted that flies exposed to hypergravity throughout development would exhibit more dramatic locomotor effects at 1 ***g***, reflecting plasticity arising from development at higher gravity levels. By assessing locomotion at 1 ***g*** after hypergravity exposure, we revealed how different locomotor phenotypes vary in their sensitivity to altered gravity. Indeed, locomotion in *Drosophila* is a well-established model for studying neuromuscular performance under environmental challenges (Bellen et al., 2010; Gargano et al., 2005). Here, we examined a range of locomotor assays: spontaneous climbing, startle-induced climbing and monitoring of daily activity. Together, these behaviors provide complementary insights into how altered gravitational loads influence locomotor control, clarify how hypergravity exposure modifies locomotion post-exposure and establish a behavioral foundation for future mechanistic analyses of gravity-induced neuromuscular and metabolic adaptation.

## MATERIALS AND METHODS

### *Drosophila* husbandry

Heisenberg Canton-S *Drosophila melanogaster* females (obtained from the Dickinson lab, California Institute of Technology) were used for all experiments. Flies were reared on a standard yeast and cornmeal-based diet at 25°C and 70% humidity on a 12 h:12 h light:dark cycle. To prevent water pooling during centrifugation, the food medium was boiled 10–15 min longer during preparation to ensure firmness under hypergravity. Prior to eclosion, female pupae were collected and placed in individual *Drosophila* vials with food and left to eclose and mature over a 48 h period. These same vials, containing both food and flies, were then transferred to the centrifuge for hypergravity treatment. For acute (24 h) exposure experiments, all virgin female flies were 3 days old at the start of hypergravity treatment and underwent behavioral assays at 1 ***g*** beginning at 4 days old. For chronic exposure experiments, flies were raised under hypergravity continuously from embryo to 3 days post-eclosion and similarly underwent behavioral assays at 1 ***g*** starting on day 4 of adulthood. All flies used in this study were strictly age matched. Behavioral assays were conducted within defined age windows: SING assays were performed at 4–10 days post-eclosion, spontaneous (non-startle) climbing at 4 days and daily activity monitoring at 5–11 days. The same age-matching criteria were applied to the chronic exposure experiments. Females were selected based on preliminary experiments showing greater consistency in climbing behavior over consecutive days compared with males. Experiments were conducted on individual flies to avoid confounds of social interactions in our assays.

This work did not require ethical approval from an animal welfare committee.

### Hypergravity treatment

**Fig. 1.**
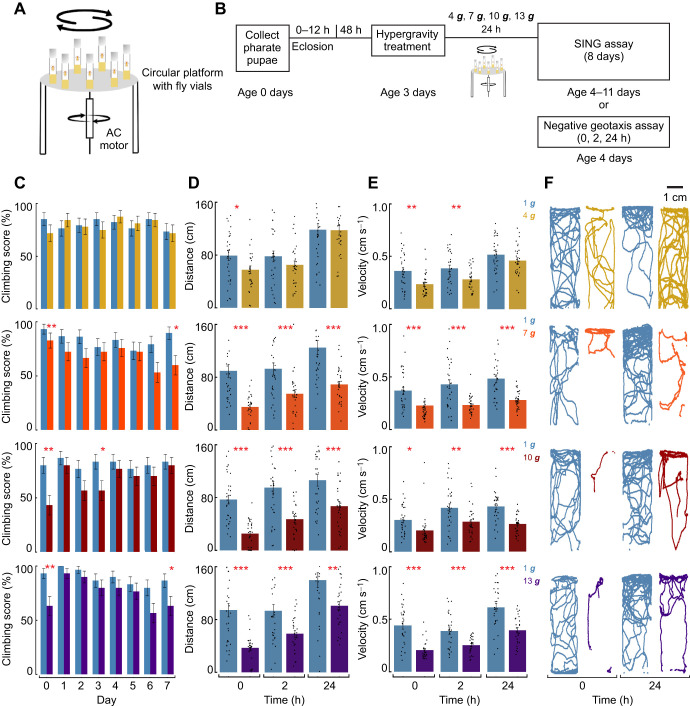
**Hypergravity induces impairment in adult *Drosophila melanogaster* negative geotactic behavior.** (A) A custom-built hypergravity simulator consisting of a circular plate attached to an AC motor via a rotating shaft. (B) Experimental timeline for assessing negative geotaxis following acute hypergravity exposure. SING, startle-induced negative geotaxis. (C) Climbing score on days 1–7, calculated as the proportion of flies that climbed 15 cm within 5 s [mean±s.e.m., generalized linear mixed model, GLMM, *n*=34 (1 ***g***), *n*=32 (4 ***g***); *n*=30 (1 ***g***), *n*=29 (7 ***g***); *n*=30 (1 ***g***), *n*=28 (10 ***g***); *n*=30 (1 ***g***), *n*=30 (13 ***g***)]. (D) Total distance moved within 5 min in the absence of startle stimuli at 0, 2 and 24 h post-hypergravity treatment [means±s.e.m., mixed-effects model, *n*=30 (1 ***g***); *n*=29 (4 ***g***); *n*=29 (7 ***g***); *n*=30 (10 ***g***); *n*=28 (13 ***g***)]. (E) Mean velocity of flies over 5 min in the absence of startle stimuli at 0, 2 and 24 h post-hypergravity treatment [means±s.e.m., mixed-effects model, *n*=30 (1 ***g***); *n*=29 (4 ***g***); *n*=29 (7 ***g***); *n*=30 (10 ***g***); *n*=28 (13 ***g***)]. For C–E: 1 ***g*** control flies (blue), 4 ***g*** (yellow), 7 ***g*** (red), 10 ***g*** (burgundy) and 13 ***g*** (purple). (F) Representative individual trajectories for flies in the negative geotaxis assay in the absence of startle stimuli at 0 and 24 h post-hypergravity treatment [individual control (blue) and hypergravity-exposed (other colors)]. Data represent biologically independent samples from three trials. All statistical tests were two-tailed. False discovery rate (FDR) correction was applied for multiple comparisons. Asterisks denote statistically significant differences between hypergravity-exposed flies and 1 ***g*** controls (*P<0.05; **P<0.01; ***P<0.001).

Gravity-level manipulation was accomplished using a custom-built hypergravity centrifuge, as described in previous studies (Hateley et al., 2016; Hosamani et al., 2016; Lints and Le Bourg, 1989; Mhatre et al., 2022; Schilder and Raynor, 2017). In brief, a 2-horsepower (≈1.49 kW) permanent magnet AC motor was connected to a rotating shaft via a system of pulleys and a pulley belt. A 30 cm diameter circular platform was affixed to the shaft, permitting the placement of fly vials along its periphery. A variable speed controller, connected to the motor, facilitated adjustments to different simulated gravitational levels [4 ***g*** (158 rpm), 7 ***g*** (202 rpm), 10 ***g*** (245 rpm), 13 ***g*** (278 rpm)] (Fig. 1A). Reported ***g*** levels refer to centrifugal acceleration only, and because centrifugal acceleration and Earth's 1 ***g*** gravitational acceleration are orthogonal, the resultant gravity forces experienced by the flies are only minimally higher [e.g. √(4^2^+1^2^)≈4.1 ***g***; √(13^2^+1^2^)≈13.03 ***g***]. Thus, for simplicity, we refer to each ***g***-level by its closest integer value. The simulator was placed inside an incubator to conduct experiments under controlled environmental conditions at 25°C and 60% humidity on a 12 h light:12 h dark cycle.

Experimental flies were subjected to either acute (24 h) or chronic (∼10 days) hypergravity exposure. The chronic treatment encompassed the entire *D. melanogaster* developmental lifecycle from egg to adult. Developmental timing under hypergravity did not differ noticeably from that of 1 ***g*** controls, with pupation and eclosion consistently occurring at ∼9–10 days at 25°C. We changed food vials every generation (∼9–10 days) during chronic exposure. Although food pooled to one side at 4 ***g*** and 7 ***g*** during chronic hypergravity exposure, we consistently obtained enough pupae to conduct the assays. Briefly, parents were allowed to mate in hypergravity and then removed after 48 h. The flies developed from egg to adult in hypergravity for ∼10 days (first generation) and were maintained in hypergravity until tested. Behavioral assays were performed at 3 days post-eclosion. For tenth generation flies, each generation was allowed to develop fully to adulthood under hypergravity, with offspring of each cross haphazardly chosen as parents for the next generation. Parents were all 2 days old at pairing, and after 10 days the newly eclosed offspring of these matings were paired for the next generation. All control flies (1 ***g***) were reared and maintained beside the simulator for the entire experiment to ensure identical environmental conditions, as is standard in hypergravity studies (Hosamani et al., 2016; Le Bourg and Lints, 1992; Lints and Le Bourg, 1989; Schilder and Raynor, 2017), but were never exposed to hypergravity. For each dataset, we ran separate controls independently, including for the duration of the multigenerational experiments. To ensure that rotational effects alone did not influence behavior, we conducted a pilot experiment in which flies were rotated at 57 rpm (equivalent to 1 ***g***) without hypergravity. This pilot produced no significant differences in climbing or locomotor activity compared with non-rotated controls. Therefore, in all experiments reported in this paper, 1 ***g*** control flies were maintained without rotation, positioned directly beside the simulator to ensure identical environmental conditions.

During our initial first generation chronic hypergravity exposure experiment, water pooling led to drowning in some experimental flies, which were subsequently discarded from analysis. However, control flies were not affected and were retained, leading to an unequal sample size between the experimental (4 ***g***, 7 ***g***) and control (1 ***g***) groups in the chronic data. This issue was resolved in subsequent experiments by extending the boiling time of the food medium during preparation, as described above.

### Behavioral assays

#### Negative geotaxis assays

Following hypergravity exposure, flies underwent two types of negative geotaxis assays. Both assays were initiated 10 min after removal from the centrifuge. For startle-induced negative geotaxis (SING), individual flies were placed in standard fly vials (95 mm in length, 25 mm in diameter) on a custom-built acrylic tap-down apparatus, or ‘swing apparatus’. This device ensured all vials were tapped to the bottom simultaneously with equal force (Fedele et al., 2014), as is standard for many geotaxis assays. A custom-built setup to record climbing was built with an array of 850 nm LEDs, diffused by a translucent sheet, provided backlighting for a front-mounted camera (Blackfly, FLIR) recording at 37 frames s^−1^. To induce climbing, the platform was raised for 5 s and then released, instantly tapping the flies to the bottom of the vials. The flies' response was recorded for a minimum of 900 frames (25 s). Videos were analyzed using Ethovision XT (Noldus, Inc.), which automatically detected and tracked the centroid *x*–*y* position of each fly in each frame. Locomotor parameters were computed from these positional data. Climbing performance in the SING assay was quantified as the proportion of flies that crossed the 5 cm mark within 15 s (Kamikouchi et al., 2009; Mhatre et al., 2022; Sun et al., 2009). Flies were kept in their original vials, and never experienced anesthesia in these assays.

To assess flies' negative geotaxis and climbing behavior in the absence of a startle stimulus, we conducted experiments without tapping down flies. Individual flies were removed from the simulator and recorded for 5 min in the dark following hypergravity exposure, allowing observation of their spontaneous negative geotaxis behavior. We recorded their distance, velocity, trajectories, number of transitions to the top to the bottom of the vial and transitions from the bottom to the top of the vial as Ethovision XT outputs. Tortuosity was calculated as the ratio of total path length to straight-line distance. Tortuosity was calculated from 2D trajectories and did not account for the curvature of the cylindrical vial; however, this limitation applies equally across all groups and therefore does not affect relative comparisons. To prevent dehydration, we added 70 µl of distilled water to the fly food daily after flies were recorded in both sets of experiments. Flies were never anesthetized in these assays, and each fly was tested only once per day. Each assay involved a single trial per fly per day (i.e. no technical replicates were performed), and *n* always refers to the number of individual flies. Further, each fly was assigned to only a single behavioral assay – SING, negative geotaxis without startle or locomotor activity monitoring.

#### Locomotor activity recording

Adult female *D. melanogaster*, aged 3–4 days, were individually recorded in DAM5H multibeam Drosophila Activity Monitors (DAMs) (Trikinetics, Waltham, MA, USA) under standard 12 h light:12 h dark conditions, with light intensities ranging from 60 to 100 lx at 25°C. After hypergravity exposure, the flies were briefly CO_2_ anesthetized and loaded into borosilicate glass tubes (80 mm in length, 5 mm in diameter) containing 2% agarose and 5% sucrose at one end, with the other end plugged with cotton (Chiu et al., 2010; Sullivan, 2002). DAM tubes were positioned horizontally (perpendicular to the Earth's gravity vector) during recording. All flies were given 8–12 h to acclimate to the DAMs. The DAM5H system utilizes 15 infrared (IR) beams that bisect each glass tube, spaced at 3 mm intervals. Fly activity was measured by counting the breaks of one or more IR beams, with activity counts recorded every minute. The flies' locomotor patterns were analyzed using the open-source data analysis software ShinyR-DAM (Cichewicz and Hirsh, 2018). Activity patterns were separated into day (ZT0–ZT12 or 05:00 h to 17:00 h; ZT0=lights on) and night (ZT12–ZT24 or 17:00 h to 05:00 h; ZT12=lights off), where zeitgeber time (ZT) indicates time relative to environmental light cues (Aschoff, 1965). Mean locomotor activity per minute was calculated as the average number of beam crosses per minute, computed across all flies over 7 days for each hour at each gravity level. Morning and evening activity peaks were quantified by averaging locomotor activity per minute across flies during the hour before lights on (05:00 h) and the hour before lights off (17:00 h), averaged over 7 days, at each gravity level. Total daily activity was measured as the cumulative number of activity counts per day, averaged across flies over 7 days at each gravity level. Day and night activity was calculated as the average locomotor activity per minute, per fly, during the light (day) and dark (night) phases, computed across 7 days for each gravity level. Data were excluded from analysis if the incubator's humidity dropped below 50%, if the temperature was above 25°C or below 24°C (which occurred twice during the study), or if flies were considered dead, defined as showing fewer than 100 activity counts per day. A total of 15 flies were considered dead out of 677 tested across all of our activity assays.

#### Measuring long-term behavioral effects following acute hypergravity exposure

To determine whether 24 h of hypergravity exposure induced behavioral effects that persisted beyond 1 week, we measured spontaneous climbing behavior and activity throughout the lifespan of 4 ***g*** and 7 ***g***-exposed flies. As above, 3 day old adult female flies were exposed to hypergravity at 4 ***g*** or 7 ***g*** for 24 h. Following exposure, flies were returned to 1 ***g*** and maintained on standard food for longitudinal behavioral assessment. Flies were transferred to fresh food every 7 days throughout the experiment. We performed negative geotaxis assays without startle at 4, 21, 38 and 55 days of age, thus measuring behavior up to 69% of 7 ***g***-exposed flies' 80 day lifespans. At each time point, individual flies were recorded for 5 min in the dark at 1 ***g***, allowing observation of spontaneous negative geotaxis behavior as described for the short-term assays (above). To quantify long-term locomotor activity following acute hypergravity exposure, a separate cohort of flies was used for DAM measurements. Individual flies were loaded into DAMs and recorded continuously at 1 ***g*** for three consecutive days at 5–7, 22–24, 39–41 and 56–58 days of age, representing measurements up to 71% of 7 ***g***-exposed flies' 74 day lifespans. We elected to measure activity over 3 rather than 7 days because 3 days was sufficient for us to capture activity across our age ranges. Activity was recorded every minute and averaged into 1 h bins to visualize temporal locomotor patterns across the 24 h cycle. After each DAM recording period, flies were transferred back to fresh food vials until the next measurement.

We monitored survival in the same flies used for each behavioral assay, with separate survival curves generated for the negative geotaxis and DAM cohorts, as these experiments were conducted on independent groups of flies. We examined whether survival differed between treatment groups using Kaplan–Meier survival curves. Mortality was recorded every 2 days at 1 ***g*** until all individuals had died.

### Triacylglyceride quantification

We quantified whole-body triacylglyceride (TAG) levels, which in *Drosophila* predominantly reflect lipid stored in the fat body with minor contributions from other tissues, using a commercially available colorimetric assay kit (Cayman Triglyceride Colorimetric Assay Kit). Three-day-old adult female flies were exposed to hypergravity at 4 ***g*** or 7 ***g*** for 24 h and then transferred to food containing 2% agar and 5% sucrose until assayed. TAG measurements were performed 1 or 7 days after return to 1 ***g***.

To generate the homogenate for TAG quantification, female flies were collected using CO_2_ anesthesia, pooled in groups of five and homogenized in 100 μl of NP-40 substitute assay reagent provided with the kit. All groups were matched for age, rearing density and diet. Homogenates were centrifuged at 10,000 ***g*** for 10 min at 4°C, and the supernatant was collected. To increase lipid solubility, supernatants were heated to 100°C for 1 min and allowed to cool to room temperature with vortexing, following the manufacturer's instructions. Triglycerides were enzymatically hydrolyzed using the triglyceride enzyme mixture (Cayman Chemical, item no. 10010511), and glycerol was quantified via a coupled enzymatic reaction.

For each assay, 10 μl of fly homogenate was mixed with 150 μl of assay solution in a 96-well plate and incubated at room temperature for 60 min. We measured absorbance at 540 nm using a Synergy H1 Hybrid Multi-Mode Microplate Reader. TAG concentrations were calculated from a standard curve generated using known TAG standards and expressed as absolute TAG concentration (mg dl^−1^) per 5-fly group.

### Quantification and statistical analysis

We analyzed climbing behavior using a generalized linear mixed model (GLMM, binomial) with separate models fitted for each day, while locomotor activity (distance, velocity) and tortuosity were analyzed using a mixed-effects model with separate models fitted for each time point. In both cases, gravity was included as a fixed effect, individual variability as a random effect, and a false discovery rate (FDR) correction was applied for multiple comparisons (Benjamini and Hochberg, 1995). Fisher's exact test was used to compare transition frequencies between the top to bottom and the bottom to top of the vial between gravity groups, with an FDR correction applied. Both GLMM and mixed-effects models are robust to unequal sample sizes (Bolker et al., 2009; West et al., 2022) that arose during chronic exposure due to flies drowning. To assess recovery in negative geotaxis without startle on a within-subject basis, per-fly changes from baseline (0 h) were computed and visualized as individual spaghetti plots with group means±s.e.m. overlaid. Change-from-baseline values were analyzed using paired *t*-tests within groups and linear models to test gravity effects, with FDR correction applied for multiple comparisons.

The effects of changes in gravity on locomotor activity over time, daily activity and day/night differences were analyzed using a mixed-effects model, with trial, time and individual as random effects. *Post hoc* tests were conducted when multiple conditions were compared within a single model, requiring pairwise comparisons to identify specific differences (e.g. between different gravity conditions or between day and night), with FDR corrections applied.

Survival data were analyzed using Kaplan–Meier survival curves with an overall multivariate log-rank test to assess differences among gravity conditions (1 ***g***, 4 ***g***, 7 ***g***). Pairwise log-rank tests were performed between conditions with FDR correction applied to *P*-values. In addition, we fitted a Cox proportional hazards model with 1 ***g*** as the reference condition to estimate hazard ratios and 95% confidence intervals.

TAG levels were analyzed using a two-way ANOVA with gravity (1 ***g***, 4 ***g***, 7 ***g***) and post-exposure time point (day 1 versus day 7) as fixed factors, including their interaction. When significant effects were detected, pairwise Welch's *t*-tests were performed with FDR correction applied for multiple comparisons. TAG values are presented as means±s.e.m.

All statistical tests were performed using Python version 3.9.12 (https://www.python.org). Data visualization was conducted using the Matplotlib and Seaborn libraries. Significance levels shown in the figures refer to the corrected *P*-values obtained from statistical tests.

### Declaration of AI use

ChatGPT was used for troubleshooting Python code errors related to data plotting and for grammar checks. The authors subsequently reviewed and edited the content as necessary and take full responsibility for the publication's final content.

## RESULTS

### Hypergravity exposure induces impairment in adult *Drosophila* negative geotaxis

In *Drosophila*, locomotion can be examined through assessment of climbing behavior, where flies exhibit an innate tendency to move upward against gravity – a behavior known as negative geotaxis (Arking and Wells, 1990; Ganetzky and Flanagan, 1978; Hostetter and Hirsch, 1967; Ricker and Hirsch, 1988; Toma et al., 2002). We first sought to investigate how climbing at 1 ***g*** is affected by 24 h of hypergravity exposure. We hypothesized that exposure to increasing levels of hypergravity would lead to progressively greater impairments in climbing at 1 ***g***, as measured by changes in climbing score, velocity and/or distance moved. We exposed 3 day old female *D. melanogaster* to varying levels of hypergravity for 24 h using a custom-built centrifuge (Fig. 1A), as described in previous studies (Hateley et al., 2016; Hosamani et al., 2016; Lints and Le Bourg, 1989; Mhatre et al., 2022; Schilder and Raynor, 2017). We elected to test individual female flies to capture individual variation in responses and exclude social interactions from any of our assays. Following hypergravity treatment, we evaluated the flies' climbing abilities using the SING assay (Benzer, 1967; Inagaki et al., 2010; Rhodenizer et al., 2008; Simon et al., 2006; Sun et al., 2018) over 7 consecutive days (Fig. 1B). The SING assay is widely used to assess motor deficits associated with movement disorders and age-related locomotor decline in *Drosophila* (Aggarwal et al., 2019; Borg and Cauchi, 2013; Gargano et al., 2005; Jahn et al., 2011; Liu et al., 2015) and consists of tapping flies to the bottom of a vial and quantifying their climbing behavior*.* At 4 ***g***, flies exhibited no climbing impairment (Movies 1 and 2). However, at higher gravity levels (7 ***g***, 10 ***g***, 13 ***g***), a smaller proportion of flies climbed 5 cm in 15 s [Fig. 1C; GLMM: 7 ***g*** on day 0 (*P=*0.003) and day 7 (*P=*0.05); 10 ***g*** on day 0 (*P=*0.007) and day 3 (*P=*0.029); 13 ***g*** on day 0 (*P=*0.002) and day 7 (*P=*0.049), all *P*-values FDR corrected; Movies 3 and 4].

To facilitate comparison with a prior study that assessed startle-induced negative geotaxis in grouped flies following hypergravity exposure (Schilder and Raynor, 2017), we also tested grouped females after 13 ***g*** exposure. Grouped flies showed more pronounced SING impairment while preserving the same overall pattern (Fig. S1). These findings suggest that increased intensity of hypergravity exposure induces mild impairment in startle-induced climbing behavior.

Next, we sought to determine how the flies' negative geotaxis behavior changes in the absence of the tapping stimulus, as spontaneous locomotion and startle-induced climbing are distinct behavioral systems regulated differently (Connolly, 1967; Martin et al., 1999; Meehan and Wilson, 1987; O'Dell and Burnet, 1988). Notably, SING performance declines with age (Le Bourg and Lints, 1992; Ganetzky and Flanagan, 1978; Grotewiel et al., 2005; Jones and Grotewiel, 2011; Sun et al., 2018; Vaccaro et al., 2017), whereas spontaneous locomotion measured on horizontal surfaces tends to remain stable or even increase across much of adult life, as reflected in measures of walking activity, exploratory behavior and distance traveled (White et al., 2010). Because our assay measures spontaneous locomotion on a vertical surface, studying startle-induced and startle-independent climbing behavior, which are controlled by different neural circuits (Dickinson, 2006; LeBeau et al., 2005; Marder, 2012; Su and Wang, 2014), provides a more complete picture of how locomotion on a vertical surface responds to rapidly changing environments, such as under altered gravity. Without the additional startle stimulus, flies traveled shorter distances and moved more slowly at 4 ***g*** immediately after hypergravity exposure (0 h, *P=*0.041, mixed-effects model, all *P*-values FDR corrected) but showed recovery by 24 h at 1 ***g*** (Fig. 1D,E). After higher gravity exposures at 7 ***g***, 10 ***g*** and 13 ***g***, hypergravity-treated flies climbed more slowly and covered less distance when tested at 1 ***g*** (Fig. 1D,E). However, following exposure to these higher gravity levels, effects persisted across all time points – 0, 2 and 24 h post-hypergravity treatment [Fig. 1D,E; Movies 5 and 6, mixed-effects model, 7 ***g***, 10 ***g***: 0 h, 2 h, 24 h (*P<*0.001); 13 ***g***: 0 h, 2 h (*P<*0.001), 24 h (*P=*0.002), all *P*-values FDR corrected]. At 0 h, immediately after exposure, hypergravity-treated flies (4 ***g***, 7 ***g***, 10 ***g*** and 13 ***g***) were significantly more likely to fail to move 5 cm or more compared with 1 ***g*** flies in both the SING assay (34.7% versus 12.1%, *P*<0.001, Fisher's exact test) and the negative geotaxis assay without a startle (16.2% versus 0%, *P*<0.001, Fisher's exact test). Because SING and spontaneous negative geotaxis assays differ in duration and context, total distance moved is not directly comparable; however, changes in average velocity from 0 to 24 h were similar across assays (linear model on change in velocity, gravity×assay interaction, *P*=0.942; Fig. S2A–D). Over the course of 24 h, both experimental and control flies increased in distance traveled and velocity (Fig. 1D,E). Nevertheless, flies that experienced hypergravity at 7 ***g*** or above never recovered to control levels for either locomotion metric after 24 h. To determine whether group-averaged trends reflected within-fly locomotor changes, we analyzed per-fly changes from baseline (0 h) in distance and velocity (Fig. S3A,B). This change-based analysis showed the same graded, gravity-dependent pattern observed in averaged data: recovery was intact at 4 ***g*** despite differences in magnitude, attenuated at 7 ***g*** and 10 ***g*** for distance (linear mixed-effects models, change in distance; 7 ***g***: *P*=0.005; 10 ***g***: *P*=0.028), and was strongly impaired at 13 ***g***, where gravity significantly affected both change in distance and change in velocity (both *P*<0.001). Without startle, flies moved significantly more slowly compared with controls, and their movement trajectories further illustrated this reduction in activity (Fig. 1F).

**Fig. 2.**
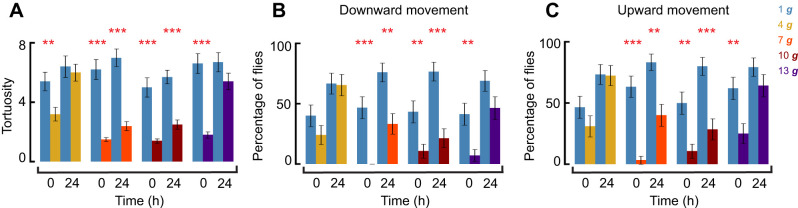
**Quantification of fly trajectories during negative geotaxis in the absence of startle.** (A) Movement tortuosity (ratio of total path length to straight-line distance) of flies recorded for 5 min at 0 and 24 h post-hypergravity exposure. (B) Percentage of flies that transitioned from the top to the bottom of the vial within 5 min at 0 and 24 h post-hypergravity exposure (Fisher's exact test). (C) Percentage of flies that transitioned from the bottom to the top of the vial within 5 min at 0 and 24 h post-hypergravity exposure (Fisher's exact test). In all panels, data are means±s.e.m. [mixed effects model; *n*=30 (1 ***g***); *n*=29 (4 ***g***); *n*=29 (7 ***g***); *n*=30 (10 ***g***); *n*=28 (13 ***g***)]. Data represent biologically independent samples from three trials. All statistical tests were two-tailed. FDR correction was applied for multiple comparisons. Asterisks denote statistically significant differences between hypergravity-exposed flies and 1 ***g*** controls (**P<0.01; ***P<0.001).

Next, we analyzed the same flies and trajectory data from Fig. 1D–F to quantify tortuosity and direction-specific walking behavior, as shown in Fig. 2. Trajectories revealed a reduction in overall path complexity at higher gravity levels, as reflected by lower tortuosity (ratio of total path length to straight-line distance) with increasing gravity level exposure (Fig. S4A,B; Fig. 2A; mixed-effects model, 4 ***g***, 0 h: *P=*0.004; 7 ***g***, 10 ***g***, 0 h, 24 h: *P*<0.001; 13 ***g***, 24 h: *P*<0.001; all *P*-values FDR corrected). Additionally, a significantly smaller proportion of flies successfully transitioned from the top to the bottom of the vial (Fig. 2B, downward movement, Fisher's exact test, 7 ***g***, 0 h: *P<*0.001; 24 h: *P=*0.0016; 10 ***g***, 0 h: *P=*0.007; 24 h: *P<*0.001; 13 ***g***, 0 h: *P=*0.007; 24 h: *P*>0.05; all *P*-values FDR corrected) and from the bottom to the top of the vial at higher gravity levels compared with controls (Fig. 2C, upward movement, Fisher's exact test, 7 ***g***, 0 h: *P<*0.001; 24 h: *P=*0.0016; 10 ***g***, 0 h: *P=*0.003; 24 h: *P<*0.001; 13 ***g***, 0 h: *P=*0.007; 24 h: *P*>0.05; all *P*-values FDR corrected), with similar effects for both upward and downward movement (binomial test, all *P*>0.05). Path analyses showed larger immediate locomotor changes after the return to 1 ***g*** in flies previously exposed to 7–13 ***g*** compared with 4 ***g***-exposed flies. At 24 h, 7 ***g***- and 10 ***g***-treated flies showed no recovery, whereas tortuosity and directional movement did not differ between 1 ***g***- and 13 ***g***-treated flies. However, 13 ***g*** flies still moved more slowly and covered less distance than their 1 ***g*** controls. These results show reduced vertical locomotion after hypergravity exposure, affecting both upward and downward movement comparably (Figs 1F and 2).

### Hypergravity exposure modulates daily activity in adult *Drosophila*

**Fig. 3.**
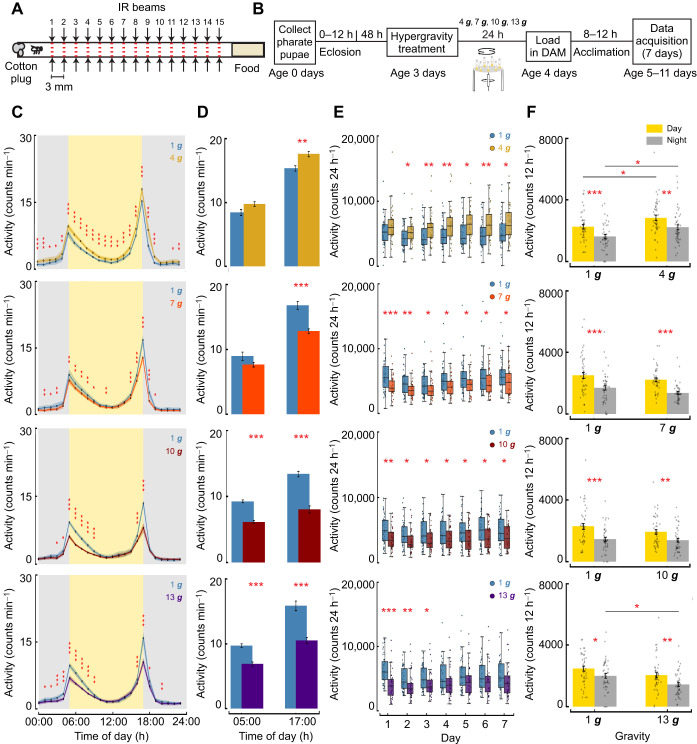
**Hypergravity modulates daily activity in adult *D. melanogaster*.** (A) Drosophila Activity Monitor tube with 15 infrared (IR) beams. (B) Experimental design for activity assay after acute hypergravity exposure. (C) Mean locomotor activity per minute averaged across flies and days at every hour for each gravity level (means±s.e.m., mixed-effects model). Control (1 ***g***) flies are shown in blue, and other colors represent different hypergravity levels as indicated. (D) Mean activity per minute binned 1 h before lights on (05:00 h) and 1 h before lights off (17:00 h) averaged across flies and days at each gravity level (means±s.e.m.). (E) Total daily activity count averaged across flies for each day (mixed-effects model). Boxplots indicate 50% of values for a given gravity level contained within the box (interquartile range, IQR), with whiskers extending to 1.5 times the IQR. Data points are plotted individually, and the median is marked by a thick line. (F) Mean activity count per fly for lights on (day) and lights off (night) averaged across the experiment duration (means±s.e.m., mixed-effects model). For C–F, *n*=48 (1 ***g***), *n*=46 (4 ***g***), *n*=44 (7 ***g***), *n*=40 (10 ***g***), *n*=40 (13 ***g***). Data represent biologically independent samples from three trials. All statistical tests were two-tailed. FDR correction was applied for multiple comparisons. Asterisks denote statistically significant differences between hypergravity-exposed flies and 1 ***g*** controls (*P<0.05; **P<0.01; ***P<0.001).

Our results, derived from examining climbing behavior with and without a startle stimulus, indicate that adult female *D. melanogaster* exhibit impaired locomotion under hypergravity. To further investigate the effect of hypergravity exposure on daily activity patterns, we used DAMs for activity monitoring over the course of 7 days at 1 ***g*** under typical 12 h:12 h light:dark conditions. We hypothesized that hypergravity exposure would also impact locomotor patterns by decreasing activity levels, with larger effects after exposure to higher gravity levels. Three-day-old *D. melanogaster* females were exposed to acute hypergravity treatment and then loaded into individual DAM tubes at 1 ***g*** (Fig. 3A). We recorded activity every minute over a 24 h period for 7 consecutive days at 1 ***g*** (Fig. 3B). To assess whether hypergravity exposure led to changes in activity throughout the course of 24 h, we averaged data across the 7 days and binned them into 1 h intervals. All control and experimental flies exhibited the typical daily activity pattern, with two activity peaks, a midday siesta and a night-time rest period, consistent with previous studies (Chiu et al., 2010; Dubowy and Sehgal, 2017; Sullivan, 2002; Tataroglu and Emery, 2014). Flies exposed to 4 ***g*** exhibited increased activity throughout the day (Fig. 3C; mixed-effects model, *P*-values indicated in figure, all *P*-values FDR corrected). However, after 7 ***g*** exposure, activity levels decreased relative to controls. After 10 ***g*** and 13 ***g*** treatment, activity levels decreased further, with the most significant reduction observed during the activity peaks (Fig. 3C,D; mixed-effects model; 4 ***g*** at 05:00 h: *P=*0.0015; 7 ***g*** at 17:00 h: *P<*0.001; 10 ***g***, 13 ***g*** at 05:00 h and 17:00 h: all *P<*0.001; all *P*-values FDR corrected). As gravity levels increased, activity after return to 1 ***g*** changed primarily during peak periods rather than uniformly across the 24 h cycle. Strikingly, flies exposed to 4 ***g*** showed increased activity, whereas higher gravity levels reduced activity, with effects persisting for up to 1 week.

When we examined how activity changed over the course of the 1 week trial, we found increases in overall activity from day 2 to day 7 for 4 ***g*** and a decrease in overall activity on all days for 7 ***g*** and 10 ***g***. The 13 ***g***-treated flies showed significantly lower activity than controls for days 1–3; however, from day 4 onward, activity levels were more variable in both control and experimental flies, resulting in no statistically significant differences between groups for days 4–7 (Fig. 3E). Nevertheless, 13 ***g***-exposed flies consistently had lower activity than controls on all days. Next, we analyzed daytime and night-time activity across all gravity levels and observed that 4 ***g*** hypergravity-treated flies exhibited higher activity during both lights on and lights off periods (Fig. 3F; mixed-effects model, day and night: both *P=*0.03; all *P*-values FDR corrected). However, at 7 ***g*** and 10 ***g***, we did not find overall reductions in activity when averaging individual fly activity across 7 days and 7 nights. In contrast, after 13 ***g*** exposure, flies were less active during the night (mixed-effects model, *P=*0.015, FDR corrected), likely driven by a reduction in activity in the few hours before lights on and after lights off. Differences between the two analyses arise because the temporal analysis (Fig. 3C) averages activity by hour across flies, whereas the aggregated day/night data (Fig. 3F) preserve individual fly variability. Together, these findings show that activity levels varied with gravity intensity, but overall circadian patterns remained similar across groups. These effects were gravity-level dependent, with increased activity at a lower gravity level (4 ***g***), followed by decreased activity at higher gravity levels (7 ***g*** and 10 ***g***). Activity levels for flies exposed to 13 ***g*** were similar to those of flies exposed to 10 ***g***.

**Fig. 4.**
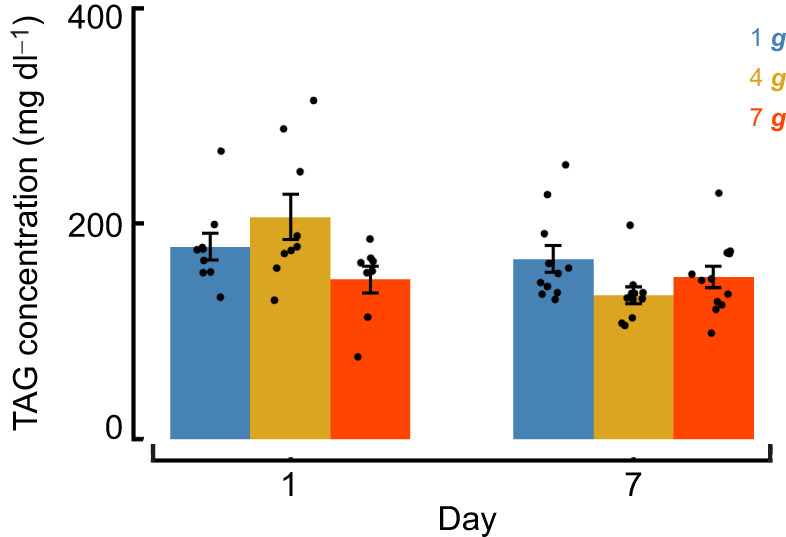
**Whole-body triacylglyceride levels vary with time after acute hypergravity exposure.** Triacylglyceride (TAG) content measured at day 1 and day 7 following return to 1 ***g*** in flies previously exposed to 1 ***g***, 4 ***g*** or 7 ***g*** (means±s.e.m.; *n*=8–12 biological replicates per group). TAG levels were quantified using a colorimetric assay, with each biological replicate consisting of a pooled sample of five flies. Data were analyzed by two-way ANOVA with gravity and day as factors. We detected a significant main effect of day (*P=*0.011) and a significant day×gravity interaction (*P=*0.011), with no main effect of gravity (*P=*0.23). Each point represents an independent biological replicate. Statistical tests were two-tailed with FDR correction applied.

To determine whether the persistent changes in daily activity following acute hypergravity exposure were accompanied by alterations in energy storage, we measured whole-body TAG levels at day 1 and day 7 after return to 1 ***g*** (Fig. 4). A two-way ANOVA revealed a significant main effect of day (*P*=0.011) and a significant day×gravity interaction (*P*=0.011), indicating that TAG levels changed over time in a gravity-dependent manner. However, there was no significant main effect of gravity alone (*P*=0.23). Although TAG levels in 4 ***g-***exposed flies were higher at day 1 and lower at day 7 relative to controls, *post hoc* comparisons between groups and across days did not remain significant after FDR correction (before FDR: *P*=0.008; after FDR: *P*=0.07). Together, these results suggest modest, time-dependent changes in lipid storage following acute hypergravity exposure, without strong evidence for sustained depletion at the time points examined.

### Acute hypergravity exposure produces long-lasting, gravity-level-dependent locomotor alterations

**Fig. 5.**
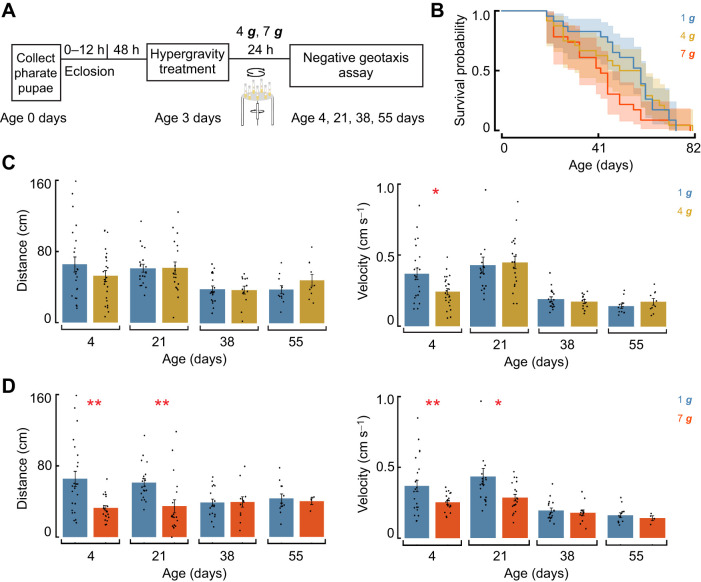
**Persistent effects of acute hypergravity exposure on negative geotaxis.** (A) Experimental design for longitudinal assessment of negative geotaxis without startle across the lifespan following acute hypergravity exposure. (B) Survival following acute hypergravity exposure. Kaplan–Meier survival curves are shown for 1 ***g*** (*n*=23), 4 ***g*** (*n*=22) and 7 ***g*** (*n*=23). Survival did not differ significantly between groups (overall log-rank *P=*0.25; all FDR-corrected pairwise comparisons non-significant). (C,D) Total distance moved and mean velocity during 5 min for flies previously exposed to 4 ***g*** and 7 ***g***, respectively, at ages 4, 21, 38 and 55 days (means±s.e.m., mixed-effects model). Data represent biologically independent samples from one trial. All statistical tests were two-tailed. FDR was applied for multiple comparisons. Asterisks denote statistically significant differences between hypergravity-exposed flies and 1 ***g*** controls (*P<0.05; **P<0.01).

To determine whether the locomotor effects of acute hypergravity exposure persist beyond the time frames assessed in our acute experiments, we performed a longitudinal study in which we exposed flies to 4 ***g*** or 7 ***g*** and repeatedly evaluated their behavior at 1 ***g*** throughout their lifetime. First, we assessed spontaneous negative geotaxis from early adulthood through to late life (Fig. 5A). Climbing performance in negative geotaxis assays typically declines with age in *Drosophila*, and evaluating this behavior across the lifespan allowed us to determine whether hypergravity alters this trajectory (Gargano et al., 2005; Jones and Grotewiel, 2011; Rhodenizer et al., 2008). Survival analysis using Kaplan–Meier curves and log-rank tests revealed no significant differences in lifespan among 1 ***g***, 4 ***g*** and 7 ***g*** flies (Fig. 5B; log-rank *P*=0.25), indicating that acute hypergravity exposure did not affect overall survival. At 4 ***g***, flies exhibited a small but significant reduction in velocity at day 4 (Fig. 5C; mixed-effects model, *P*=0.0128, FDR corrected), but no differences in distance traveled at any age and no differences in either metric at days 21, 38 or 55 (all *P*>0.05, FDR corrected). In contrast, 7 ***g*** exposure produced pronounced early-life impairments: 7 ***g***-treated flies traveled shorter distances and moved more slowly at day 4 (Fig. 5D; distance: *P*=0.00054; velocity: *P*=0.0043) and day 21 (distance: *P*=0.0021; velocity: *P*=0.0185; mixed-effects model, all *P*-values FDR corrected). By days 38 and 55, no significant differences were observed between 1 ***g***, 4 ***g*** and 7 ***g*** flies (all *P*>0.05), indicating that these early deficits did not continue to diverge with age. Comparing this independent dataset at an early time point (0 h) as part of the long-term effects analysis yielded results indistinguishable from those obtained in the initial short-term experiments for both 4 ***g*** and 7 ***g*** (Fig. 1D,E versus Fig. 5C,D; Welch's *t*-tests, FDR corrected; all *P*>0.05).

**Fig. 6.**
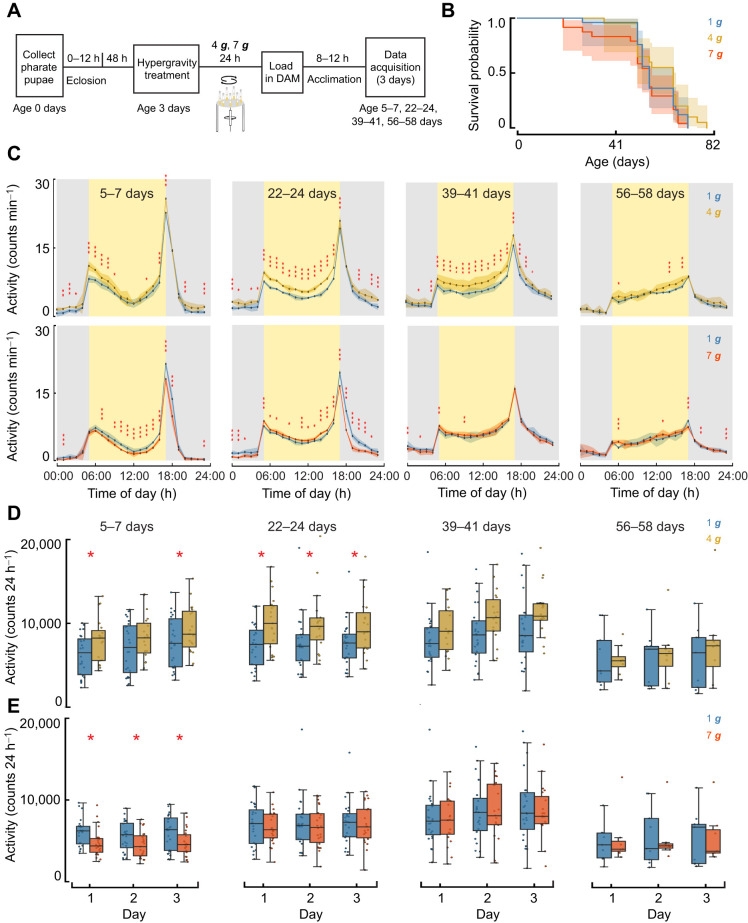
**Persistent effects of acute hypergravity exposure on daily locomotor activity.** (A) Experimental design for longitudinal monitoring of daily locomotor activity across the lifespan following acute hypergravity exposure. (B) Kaplan–Meier survival curves for flies maintained at 1 ***g*** (*n*=24), 4 ***g*** (*n*=19) and 7 ***g*** (*n*=24). No significant differences in survival were detected (log-rank test: *P=*0.30). (C) Long-term daily activity patterns for flies previously exposed to 4 ***g*** and 7 ***g***. Mean locomotor activity per minute averaged across flies and days at every hour for ages 5–7, 22–24, 39–41 and 56–58 days (means±s.e.m., mixed-effects model). (D,E) Total daily activity count for 4 ***g***- and 7 ***g***-treated flies, respectively, averaged across flies for each day for ages 5–7, 22–24, 39–41 and 56–58 days (means±s.e.m., mixed-effects model). Box plots are as described in Fig. 3E. Data represent biologically independent samples from one trial. All statistical tests were two-tailed. FDR correction was applied for multiple comparisons. Asterisks denote statistically significant differences between hypergravity-exposed flies and 1 ***g*** controls (*P<0.05).

We next evaluated whether hypergravity-induced changes in daily locomotor activity persisted across the lifespan using a longitudinal DAM5H assay at ages 5–7, 22–24, 39–41 and 56–58 days (Fig. 6A). Activity was recorded every minute for 3 consecutive days at 1 ***g*** and averaged into 1 h bins to visualize temporal locomotor patterns across the 24 h cycle. Kaplan–Meier survival analysis showed no significant differences in lifespan among the 1 ***g***, 4 ***g*** and 7 ***g*** groups in the DAM assay flies (Fig. 6B; log-rank *P*=0.30), similar to the negative geotaxis assay. At ages 5–7 and 22–24 days, 4 ***g***-exposed flies exhibited sustained hyperactivity across much of the daytime period (Fig. 6C; mixed-effects model; *P*-values indicated in figure, FDR corrected), particularly during and between the major activity peaks. This overall pattern is consistent with reports that baseline spontaneous activity can vary with aging and genetic background in some fly cohorts (Thiem et al., 2024). Hyperactivity in 4 ***g***-treated flies remained detectable at ages 39–41 days, although its magnitude declined relative to earlier ages, with significant differences restricted to specific daytime and early-night intervals (*P*<0.05, FDR corrected). By 56–58 days of age, 4 ***g***-exposed flies continued to show elevated daytime activity relative to controls, though differences were modest and confined to limited intervals, indicating progressive attenuation of the hyperactivity phenotype over time (Fig. 6C). In contrast, flies previously exposed to 7 ***g*** showed markedly reduced activity early in life (Fig. 6C; ages 5–7 days: widespread reductions throughout the daytime period, *P*<0.01, FDR corrected). By ages 22–24 days, 7 ***g*** flies continued to show lower activity, although the deficit was limited to specific daytime intervals. By ages 39–41 days, locomotor activity in 7 ***g***-treated flies largely converged with controls, with only isolated time points remaining significantly different (*P*<0.05). At ages 56–58 days, activity profiles of 7 ***g***-exposed flies overlapped almost entirely with those of 1 ***g*** controls across the 24 h cycle, with no consistent differences detected (*P*>0.05 for most time points). When total activity was aggregated across each 24 h period (Fig. 6D; mixed-effects model, FDR corrected), differences between 1 ***g***- and hypergravity-treated flies were less pronounced than in the hour-resolved profiles. This reflects the fact that increases during some portions of the day and decreases during others tend to average out when collapsed into a single daily metric, an effect noted in prior analyses of binned DAM data (Cichewicz and Hirsh, 2018). Accordingly, although 4 ***g***-exposed flies tended to exhibit higher median daily activity – particularly at earlier ages – these differences were not consistently significant across all ages measured. Analyzing the long-term DAM assay at overlapping early time points of the initial short-term experiments (days 5–7) yielded results indistinguishable from those obtained in the short-term experiments for both 4 ***g*** and 7 ***g*** (Fig. 3C,D versus Fig. 6C,D; fixed-effects linear models, FDR-corrected, all *P*>0.05).

### Chronic hypergravity exposure exacerbates locomotion impairment

**Fig. 7.**
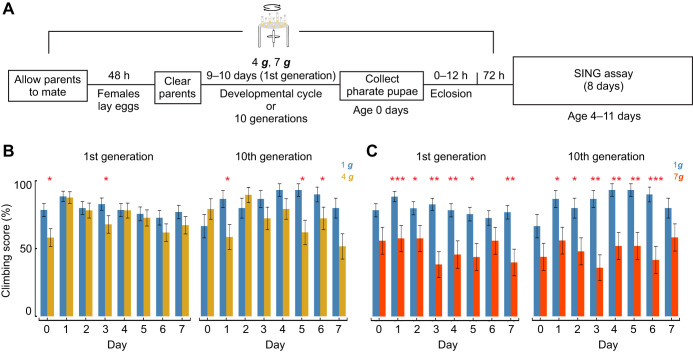
**Chronic hypergravity exposure exacerbates impairment in the SING assay.** (A) Experimental design for testing climbing after chronic hypergravity exposure. (B) Climbing scores (proportion of flies that climbed 15 cm within 5 s) for SING assay following 4 ***g*** exposure after 1 or 10 generations [mean climbing score±s.e.m., GLMM, first generation: *n*=55 (4 ***g***), *n*=70 (1 ***g***); tenth generation: *n*=29 (4 ***g***), *n*=30 (1 ***g***)]. (C) Climbing scores for chronic exposure to 7 ***g*** [first generation: *n*=25 (7 ***g***), *n*=70 (1 ***g***); tenth generation: *n*=24 (7 ***g***), *n*=30 (1 ***g***)]. When comparing acute and chronic exposure, no significant generational differences were observed at 4 ***g*** (*P=*0.095), whereas both first and tenth generation flies showed significantly greater impairment compared with acutely exposed flies at 7 ***g*** (first generation: *P=*0.014; tenth generation: *P<*0.001). First generation data represent biologically independent samples from three trials; tenth generation data are from two trials. All statistical tests were two-tailed. FDR correction was applied for multiple comparisons. Asterisks denote statistically significant differences between hypergravity-exposed flies and 1 ***g*** controls (*P<0.05; **P<0.01; ***P<0.001).

Next, we sought to understand how rearing in hypergravity modulates locomotion post-exposure as flies transition back to 1 ***g***. Female flies were chronically exposed to hypergravity (4 ***g*** or 7 ***g***) for their entire developmental cycle (first generation, 9–10 days) or across 10 generations (tenth generation), and their climbing ability and daily activity were examined post-exposure at 1 ***g*** (Fig. 7A). Control (1 ***g***) flies were reared and handled identically across generations but were never exposed to hypergravity at any stage. We hypothesized that flies reared under chronic hypergravity would exhibit greater locomotor changes upon returning to 1 ***g*** than those exposed acutely, as development under hypergravity may increase energetic demands and lead to plastic physiological changes that result in greater locomotion differences when flies return to 1 ***g***. Consistent with our hypothesis, 4 ***g*** first generation flies showed SING climbing impairments at 1 ***g***, with significantly lower climbing scores on day 0 and day 3 post-exposure (Fig. 7B; GLMM, day 0: *P=*0.019; day 3: *P=*0.046; FDR corrected) that were not observed under acute treatment (Fig. 1C). Similar to first generation flies, we observed reductions in climbing scores in 4 ***g*** tenth generation flies relative to controls (Fig. 7B; day 1: *P=*0.045; day 5: *P=*0.014; and day 6: *P=*0.037; FDR corrected). After 7 ***g*** exposure, the decrease in climbing score was more pronounced than for first generation 4 ***g***-treated flies, with significant differences observed on nearly all days post-exposure for first generation flies (Fig. 7C; first generation: day 1: *P*<0.001; day 2: *P=*0.02; day 3: *P=*0.002; day 4: *P=*0.007; day 5: *P=*0.013; day 7: *P=*0.006; FDR corrected). As with 4 ***g***-exposed flies, both first and tenth generation flies exhibited similar declines in climbing score in the SING assay at 7 ***g*** (Fig. 7C; tenth generation: day 1: *P=*0.045; day 2: *P=*0.024; day 3: *P=*0.008; day 4: *P=*0.009; day 5: *P=*0.002; day 6: *P*<0.001; FDR corrected). When comparing acute and chronic exposure, no significant generational differences were observed at 4 ***g*** (mixed-effects model, *P*>0.05). At 7 ***g***, both first and tenth generation flies showed significantly greater impairment compared to acutely exposed flies (Figs 1C and 7C; first generation: *P*=0.014; tenth generation: *P*<0.001).

**Fig. 8.**
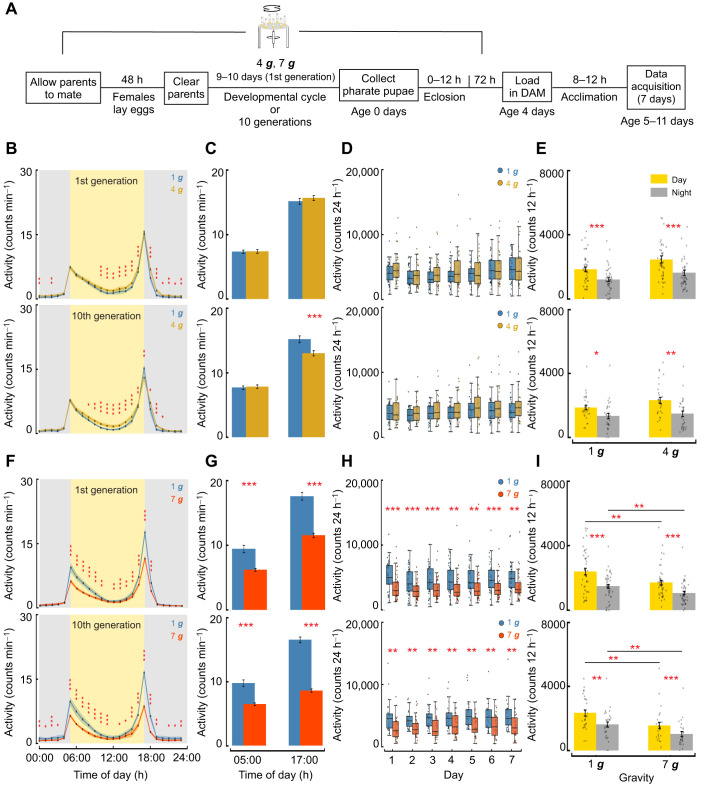
**Chronic hypergravity exposure exacerbates impairment in daily activity.** (A) Experimental design for assessing activity pattern after chronic hypergravity exposure. (B,F) Mean locomotor activity per minute for chronic exposure to 4 and 7 ***g***, respectively, averaged across flies and days at every hour for each gravity level (means±s.e.m., mixed-effects model). Control (1 ***g***) flies are shown in blue, and other colors represent different hypergravity levels as indicated. (C,G) Mean activity per minute for chronic exposure to 4 and 7 ***g***, respectively, binned 1 h before lights on (05:00 h) and 1 h before lights off (17:00 h) averaged across flies and days at each gravity level. (D,H) Total daily activity count for chronic exposure to 4 and 7 ***g***, respectively, averaged across flies for each day (means±s.e.m., mixed-effects model) for chronic exposure to 4 ***g***. Box plots are as described in Fig. 3E. (E,I) Mean activity count per fly for chronic exposure to 4 and 7 ***g***, respectively, for lights on (day) and lights off (night) averaged across the experiment duration (means±s.e.m., mixed-effects model) ***g***. First generation: *n*=48 (1 ***g***); *n*=40 (4 ***g***); *n*=43 (7 ***g***); tenth generation: *n*=32 (1 ***g***); *n*=30 (4 ***g***); *n*=27 (7 ***g***). First generation data represent biologically independent samples from three trials; tenth generation data are from two trials. All statistical tests were two-tailed. FDR correction was applied for multiple comparisons. Asterisks denote statistically significant differences between hypergravity-exposed flies and 1 ***g*** controls (*P<0.05; **P<0.01; ***P<0.001).

Next, we examined changes in activity levels after chronic exposure using DAMs, as described above (Fig. 8A). First generation 4 ***g***-exposed flies showed the hyperactivity phenotype observed for 4 ***g*** acute treatment; however, the magnitude of this effect was statistically smaller in 4 ***g*** chronic flies than in flies in our 4 ***g*** acute treatment (Figs 3C and 8B; mixed-effects model, interaction effect between gravity and length of exposure *P*<0.001). We found that the tenth generation 4 ***g*** flies exhibited a similar pattern to the first generation 4 ***g*** flies, showing hyperactivity outside of activity peaks; however, tenth generation flies showed a decline in the evening activity peak (Fig. 8C; mixed-effects model, *P*<0.001, FDR corrected). Although acutely treated 4 ***g*** flies displayed elevated total activity counts and increased activity during both the day and the night (Fig. 3E,F), chronic exposure resulted in activity levels similar to those of controls when examined over the course of 7 days or when aggregated into lights on–lights off periods, leading to no significant differences for either generation at 4 ***g*** (Fig. 8D,E).

After 7 ***g*** exposure, both the first and tenth generation flies exhibited lower locomotor activity compared with acute exposure to 7 ***g*** (Figs 3C and 8F; mixed-effects model, interaction effect between gravity and length of exposure *P*<0.001), mimicking the activity pattern observed at 10 ***g*** and 13 ***g*** acute exposure [Fig. 3C; mixed-effects model; 7 ***g*** (chronic) versus 10 ***g*** (acute): n.s., *P=*0.26; 7 ***g*** (chronic) versus 13 ***g*** (acute): *P=*0.002; all *P*-values FDR corrected]. The decreases in locomotor activity were most prominent at the activity peaks for both generations (Fig. 8G; all *P*<0.001). The total daily activity count also showed a significant decline after chronic 7 ***g*** exposure on all days post-exposure for both generations (Fig. 8H; mixed-effects model, first generation: days 1, 2, 3 and 6: *P*<0.001; day 4: *P=*0.0015; day 5: *P=*0.0024; and day 7: *P=*0.0024; tenth generation: day 1: *P=*0.0027; day 2: *P=*0.0054; day 3: *P=*0.0019; day 4: *P=*0.0081; day 5: *P=*0.0011; day 6: *P=*0.0054; day 7: *P=*0.0054; all *P*-values FDR corrected). Reductions in both daytime and night-time activity were also observed for 7 ***g***-exposed flies compared with controls for both generations (Fig. 8I; mixed-effects model, first generation: 1 ***g*** day versus 7 ***g*** day: *P=*0.0079; and 1 ***g*** night versus 7 ***g*** night: *P=*0.0079; tenth generation: 1 ***g*** day versus 7 ***g*** day: *P=*0.0031; and 1 ***g*** night versus 7 ***g*** night: *P=*0.0018; FDR corrected). This activity decline was larger after chronic exposure than after acute exposure to 7 ***g*** (Figs 3F and 8I; mixed-effects model, *P=*0.0098, FDR corrected). Together, these findings indicate that flies reared in chronic hypergravity show persistent locomotor impairments at 1 ***g***, with no evidence of recovery or further decline across generations.

## DISCUSSION

Gravity has been a constant force throughout evolution, yet its role in governing key biological processes, such as locomotion, remains relatively poorly understood. Here, we explored various aspects of female adult *D. melanogaster* locomotion, including climbing trajectories and activity levels following varied intensities and durations of hypergravity exposure. Our findings reveal that exposure to increased gravity levels impairs locomotion at 1 ***g*** in a context-dependent manner. Specifically, climbing with a startle stimulus shows mild impairments at higher gravity levels, while movement without a startle stimulus is significantly suppressed after returning to 1 ***g***, with flies exhibiting reduced climbing, as reflected in distances, velocities and trajectories. Startle-induced ‘fight or flight’ responses may temporarily mask effects on climbing after experiencing changes in gravity, as flies at 4 ***g*** show no climbing impairment when startled but exhibit slower walking and less tortuous trajectories after testing in the absence of startle. Previous studies have reported hypergravity-induced impairments in the SING assay in older (9–55 day old) flies (Le Bourg and Lints, 1992; Lints and Le Bourg, 1989), and an increased SING response in younger flies (Schilder and Raynor, 2017), both after returning flies to 1 ***g***, suggesting that hypergravity exposure does not result in a single phenotypic effect on climbing. Differences from earlier reports (Schilder and Raynor, 2017) may reflect differences in experimental design, including sex of the flies and grouped versus individual testing. Social interactions in grouped assays can alter locomotor activity by inducing competition, aggregation or courtship behaviors, which are absent when flies are tested individually (Rooke et al., 2020; Scaplen et al., 2019). When we tested grouped females, at 13 ***g***, their SING performance was even more impaired than that of individually tested females, indicating that group housing does not explain the reduced SING response observed in our study.

After higher gravity-level exposure (7 ***g***, 10 ***g***, 13 ***g***), climbing was further reduced when flies were tested at 1 ***g***, with significant decreases in climbing behavior observed both with and without startle stimuli. In our SING assays, we did find some significant differences between treatment and control flies beyond day 0 for 7–13 ***g*** treatment groups. These differences may reflect variability in 1 ***g*** controls rather than continued decline in hypergravity-treated flies; however, the immediate impairment on day 0 represents a direct and robust effect of acute hypergravity exposure. In contrast, in our negative geotaxis without startle assay, we found more persistent effects, showing that flies failed to recover normal walking speed or trajectory complexity 24 h after exposure above 4 ***g***. It is worth noting that both control and hypergravity-treated flies increased distance traveled and velocity over this period; however, this is likely due to handling effects experienced by all groups (White et al., 2010). Impairments in vertical locomotion, including movement both upward and downward, highlight the substantial impact of hypergravity exposure on these behaviors. Alternatively, flies may be less willing to move without a startle stimulus. While the SING assay preserved startle-induced climbing – possibly driven by reflexive escape circuits (Jones and Grotewiel, 2011; Sun et al., 2018) – the absence of such stimuli in spontaneous locomotion may reveal impairments in vertical movement and/or motivation to move. Furthermore, hypergravity is known to be energetically demanding (Economos et al., 1982; Le Bourg and Lints, 1989a; Minois, 2006; Richoilley et al., 1988; Thorling and Fredens, 1995), potentially lowering the energy reserves available to flies when returning to 1 ***g***. Climbing behavior, whether uphill or downhill, may be metabolically costly, prompting flies to conserve energy by moving less following hypergravity exposure. Consistent with this idea, whole-body TAG measurements revealed time-dependent changes after acute hypergravity exposure, with a significant interaction between gravity and time following return to 1 ***g***. Although *post hoc* comparisons did not reach significance after multiple-test correction, these patterns suggest that hypergravity may transiently alter lipid utilization or recovery dynamics rather than producing a uniform depletion of energy stores. Such effects may depend on the timing of measurement or require greater sampling resolution to detect robust group differences. Startle stimuli, however, may override this energy-conservation strategy, triggering immediate energy expenditure via ‘fight or flight’ responses, a mechanism absent without a startle.

Circadian rhythms and daily activity patterns are shaped by a range of environmental and physiological factors – including light–dark cycles, feeding schedules, temperature, population density, metabolic demands and gravitational forces – as shown across multiple species (Åkesson and Hedenström, 2007; Blanco-Vives and Sánchez-Vázquez, 2009; Fuller et al., 1994; Jhaveri et al., 2007; Todorović et al., 2020; Zhang et al., 2021). These factors can influence the timing, amplitude and expression of behavioral rhythms across taxa – from zebrafish to rodents, insects and primates – highlighting the adaptive flexibility of behavioral activity in response to changing environmental conditions. Here, we describe how hypergravity exposure alters these patterns in *D. melanogaster* when flies are returned to Earth's gravity. Specifically, we observed increased activity at 4 ***g*** but a decrease at higher gravity levels, particularly at 10 ***g*** and 13 ***g***. These changes were most evident at dawn and dusk activity peaks, when crepuscular *Drosophila* are most active. In lab-reared *Drosophila*, the hour before lights on and lights off is referred to as the anticipatory phase (Chiu et al., 2010; Curran et al., 2019; Dubowy and Sehgal, 2017), which leads to peak activity at lights on or lights off. Hypergravity exposure above 4 ***g*** consistently lowered fly activity at 1 ***g***, particularly the magnitude of these anticipatory phases and activity peaks, and these effects persisted for several weeks after returning to 1 ***g***. Intriguingly, these results align with studies across diverse taxa that report alterations in circadian rhythms and activity in hypergravity (Alpatov et al., 1994; Fuller, 1984; Hoban-Higgins et al., 1995). Together, these results suggest that hypergravity induces gravity-level-dependent physiological changes in locomotor regulation, the mechanisms of which await further study.

Our longitudinal assays indicate that the persistence of hypergravity effects depends strongly on gravity level. At 4 ***g***, flies exhibited elevated locomotor activity well into late adulthood, although the magnitude declined with age, consistent with evidence that spontaneous activity and locomotor trajectories can remain durably altered during aging in *Drosophila* and other systems (Gargano et al., 2005; Grotewiel et al., 2005; Iliadi and Boulianne, 2010; Rhodenizer et al., 2008). The persistence of this phenotype suggests a lasting physiological shift rather than a transient stress response, in line with studies showing that environmental or genetic perturbations can reshape locomotor aging trajectories (Piazza et al., 2009; Rhodenizer et al., 2008). In contrast, flies exposed to 7 ***g*** showed pronounced locomotor deficits early in life – across both spontaneous negative geotaxis and DAM-recorded activity – that progressively diminished with age, converging with 1 ***g*** controls. Similar patterns of partial recovery or stabilization of locomotor performance after an initial deficit have been reported in *Drosophila* following interventions such as exercise training and other genetic or environmental manipulations (Gargano et al., 2005; Iliadi and Boulianne, 2010), suggesting that higher gravity imposes a stronger acute burden from which flies can later recover.

Survival remained high across all groups, indicating that the behavioral changes induced by hypergravity exposure were not accompanied by major reductions in viability. This is broadly consistent with classic *Drosophila* hypergravity work showing that brief or moderate exposures can have relatively small effects on lifespan, whereas chronic, life-long exposure to high gravity levels tends to shorten longevity and accelerate some aging phenotypes (Le Bourg and Lints, 1989a,b; Le Bourg and Minois, 1999; Minois, 2006). Together, our findings show that acute hypergravity exposure induces locomotor alterations that can persist for weeks, but with gravity-dependent effects: lower hypergravity (4 ***g***) produces a stable hyperactive phenotype that persists for weeks after exposure, albeit with decreasing magnitude. In contrast higher hypergravity (7 ***g***) leads to early deficits followed by partial or near-complete recovery with age.

Chronic exposure further reduces climbing, particularly at higher gravity levels, and these reductions remain stable across generations without evidence of recovery or further decline. Rearing under hypergravity may induce developmentally plastic physiological changes within a single generation, similar to other chronic stressors such as nutritional restriction, ethanol exposure, hypoxia or temperature stress (Grandison et al., 2009; Robinson et al., 2012; Wingrove and O'Farrell, 1999; Zhong and Wu, 2004). Indeed, chronic hypergravity exposure throughout development in *Drosophila* induced differential expression in over 1000 genes, including multiple genes related to cellular respiration and redox (Hateley et al., 2016). Proteomic profiling identified significant alterations in proteins related to energy metabolism, stress responses and mitochondrial function (Hosamani et al., 2016). Together, these transcriptomic and proteomic shifts in response to hypergravity exposure could have important consequences for neuromuscular performance. Over multiple generations, such rearing may amplify or compound these effects, potentially leading to more extreme phenotypic changes upon return to 1 ***g***, as observed in studies of transgenerational adaptation under nutritional, thermal and hypoxic stress in *Drosophila* (Khan and Mishra, 2024; Krittika and Yadav, 2022; Schwasinger-Schmidt et al., 2012; Zhou et al., 2011). Because we found few behavioral differences between our first and tenth generation flies, locomotor changes may be constrained by the increased energetic demands of development under sustained hypergravity. Additionally, it is possible that ten generations was insufficient time to for adaptive changes to emerge.

Our observed reductions in activity and climbing could be a consequence of prioritizing energy storage over energy expenditure, particularly following exposure to higher gravity levels. Notably, we observed increasing impairment from 7 ***g*** to 10 ***g***, while 13 ***g*** produced impairments similar to those at 10 ***g*** across negative geotaxis assays and spontaneous locomotor activity measures. This consistent pattern suggests a potential plateauing effect at higher gravity levels, possibly reflecting physiological mechanisms that are not yet fully understood. Given previous findings that indicate an increase in metabolism in flies (Le Bourg and Lints, 1989a,b) and a reduction in food intake in mice (Abe et al., 2010, 2020) after hypergravity exposure, a decrease in locomotor activity with increasing gravity-level exposure may suggest a redistribution of available energy between maintenance and locomotion. Although our TAG measurements did not show significant *post hoc* effects, the significant interaction effect suggests that energy use is altered differently depending on hypergravity level, as reflected in our behavioral data. Indeed, female flies exposed to hypergravity appear to prioritize maintenance over reproduction, laying significantly fewer eggs and experiencing a delayed peak in fecundity (Lints and Le Bourg, 1989). Similarly, stressors such as temperature extremes, starvation, hypoxia and dehydration induce reductions in locomotor activity, enabling energy conservation and prioritization of survival functions (Cheuvront et al., 2003; Hochachka and Lutz, 2001; Huey and Stevenson, 1979; Sokolova et al., 2012; Sørensen et al., 2003; Wang et al., 2006). These similar phenotypic effects in response to varied stressors suggest that acute or chronic exposure to environmental stressors, including hypergravity, may invoke similar adaptive mechanisms – a concept warranting further study.

The neuroendocrine system is a promising candidate for regulating this energy usage. Day and night activity patterns are fine-tuned by endocrine signals, specifically adipokinetic hormone (AKH), an insect analog of glucagon and the neuromodulator octopamine (OA) (Pauls et al., 2021). During the day, activity is encouraged by the AKH-OA system, whereas at night, the AKH-fat body system promotes rest. Indeed, in our data, activity did not change uniformly throughout the 24 h period in response to altered gravity, suggesting effects that are related to time of day. Hypergravity may induce changes in this neuroendocrine signaling, either by increasing or decreasing it, which ultimately could affect locomotion. In summary, our findings suggest that both higher gravity levels and the duration of exposure can impact locomotion in adult *Drosophila*, perhaps by impacting energy allocation and utilization following hypergravity stress.

## Supplementary Material

10.1242/jexbio.251327_sup1 Supplementary information
