# Supplementary material for: Hypergravity exposure leads to persistent effects on geotaxis and activity in Drosophila melanogaster
Source: J Exp Biol. 2026 Apr 23;229(8):jeb251327. doi: 10.1242/jeb.251327 (PMC13143205; doi:10.1242/jeb.251327)
Supplement: Supplementary information [file jexbio-229-251327-s1.pdf]

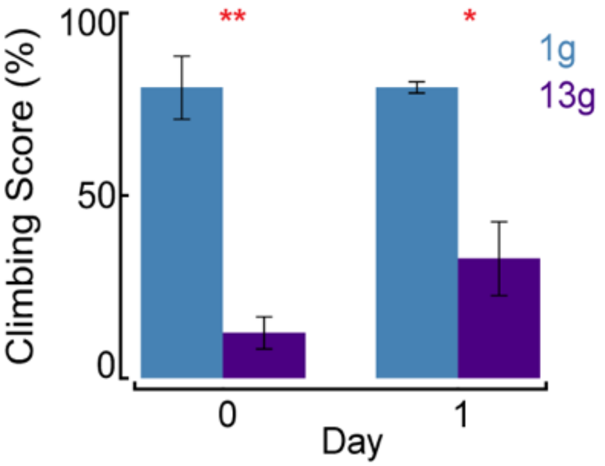

Fig. S1. SING Assay in grouped female flies. Startle-induced negative geotaxis (SING) was assessed in group assays using female flies (16 females per trial) under 1g and 13g conditions. The same cohort of flies was tested on Day 0 and Day 1. Climbing performance is shown as the percentage of flies reaching the target height per trial (mean ± SEM; n=4 independent trials per condition). Differences between gravity conditions within each day were analyzed using paired two-tailed t-tests (Day 0: 1g vs 13g, *P* = 0.0074; Day 1: 1g vs 13g, *P* = 0.0270). Asterisks denote statistically significant differences between hypergravity-exposed flies and 1g controls. **Significance levels: **P*<0.05; ***P*<0.01; ****P*<0.001.

Journal of Experimental Biology • Supplementary information

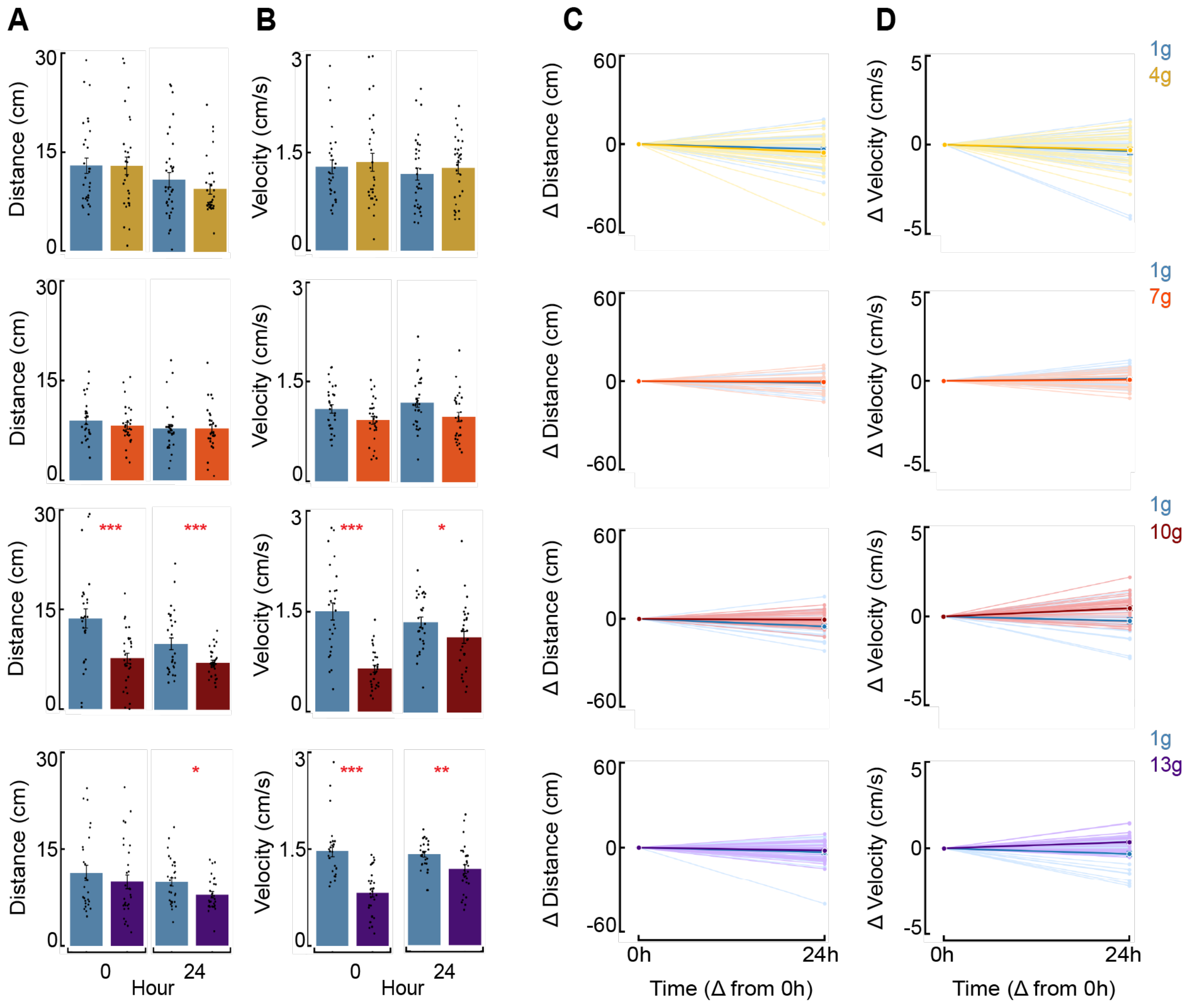

Fig. S2. Hypergravity induces impairment in adult *Drosophila* startle-induced negative geotactic (SING) behavior. (A) Total distance moved within 15 seconds in SING assay 0 and 24 hours post-hypergravity treatment (mean ± SEM, Mixed-effects model, n=34 (1g), n=32 (4g); n=30 (1g), n=29 (7g); n=28 (1g), n=30 (10g); n=30 (1g), n=30 (13g)). 1g control flies (blue), 4g (yellow), 7g (red), 10g (burgundy), 13g (purple). (B) Mean velocity of flies over 15 seconds in SING assay (mean velocity ± SEM, Mixed-effects model, n=34 (1g), n=32 (4g); n=30 (1g), n=29 (7g); n=28 (1g), n=30 (10g); n=30 (1g), n=30 (13g)). Colors as in A. (C) Change in distance from 0 to 24 hours (Δ-distance from 0 h) for individual flies assessed using the SING assay at 4g, 7g, 10g, and 13g. Thin lines represent individual flies; bold lines represent group means ± SEM. Gravity did not significantly affect Δ-distance at 4g or 7g ($P > 0.05$), but significantly reduced Δ-distance at 10g ($P = 0.003$), with no effect at 13g ($P > 0.05$). (D) Change in velocity from 0 to 24

Journal of Experimental Biology • Supplementary information

hours (Δ-velocity from 0 h) plotted as in (C). Gravity did not affect Δ-velocity at 4g or 7g ($P > 0.05$), but significantly reduced Δ-velocity at 10g ($P < 0.001$) and 13g ($P < 0.001$), indicating heightened sensitivity of startle-evoked velocity to high g-levels. Data represent biologically independent samples from three trials. All statistical tests were two-tailed. False Discovery Rate (FDR) correction was applied for multiple comparisons. Asterisks denote statistically significant differences between hypergravity-exposed flies and 1g controls. **Significance levels: *P<0.05; **P<0.01; ***P<0.001.

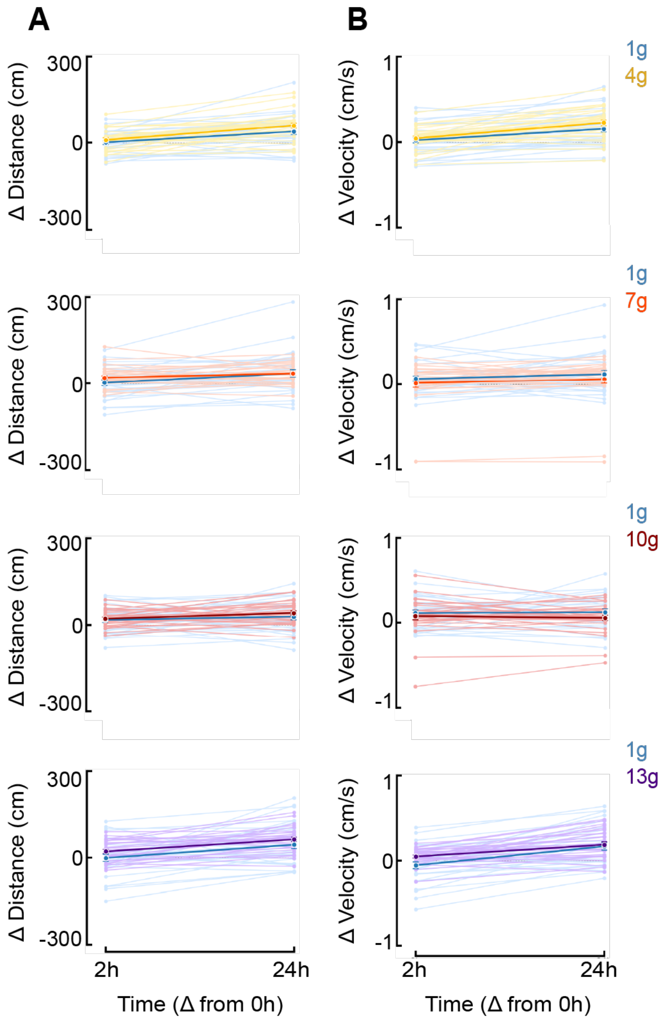

Fig. S3. Hypergravity induces impairment in adult *Drosophila* negative geotactic behavior in the absence of startle. (A) Δ-distance (change from 0 h baseline) for individual flies recorded for 5 min in the absence of startle stimuli at 4g, 7g, 10g, and 13g, measured at 2 h and 24 h post-exposure. Thin lines represent individual flies; bold lines represent group means ± SEM. Distance increased over time in both control and hypergravity-exposed flies at all g-levels. However, recovery magnitude was progressively reduced with increasing gravity, with significant gravity effects on Δ-distance at 7g (P = 0.005), 10g (P = 0.028), and 13g (P < 0.001). (B) Δ-velocity (change from 0 h baseline) plotted as in (A). Δ-velocity increased from 2 h to 24 h in control flies and in 4g-exposed flies, indicating intact time-dependent recovery at these gravity levels. In contrast, a significant gravity effect on Δ-velocity was detected only at 13g (P < 0.001), whereas no significant gravity effects on Δ-velocity were observed at 7g or 10g (P > 0.05). Data represent biologically independent samples from three trials. Statistical comparisons were performed using linear mixed-effects models and paired t-tests with false discovery rate (FDR) correction for multiple comparisons. Asterisks denote significant differences between hypergravity-exposed flies and 1g controls (P<0.05; *P<0.01; **P<0.001).

Journal of Experimental Biology • Supplementary information

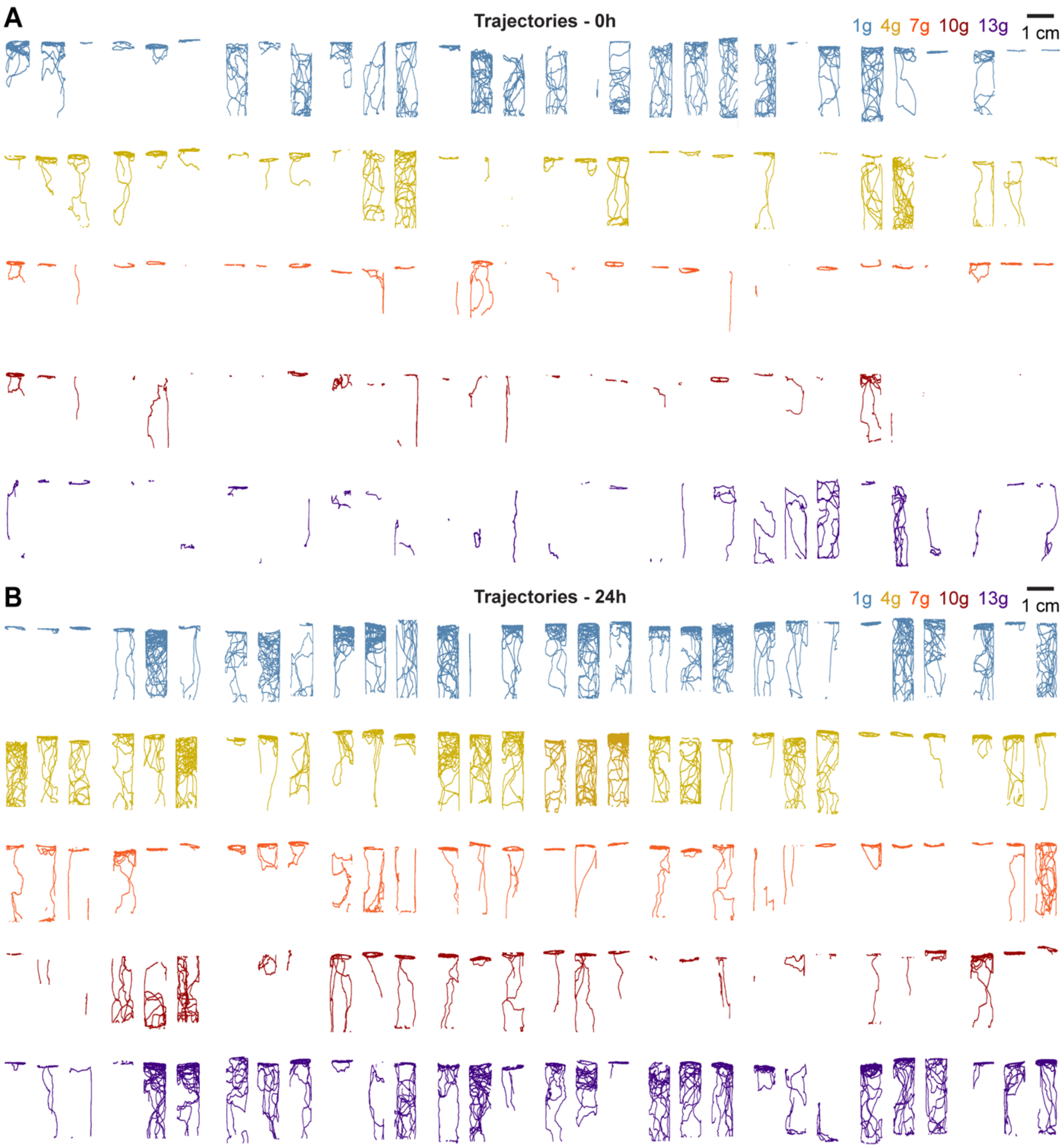

Fig. S4. Trajectories of flies recorded for 5 minutes during negative geotaxis in the absence of startle. (A) Trajectories of 1g (blue), 4g (yellow), 7g (orange), 10g (red), and 13g (purple) flies at 0 hours post-hypergravity exposure. (B) Trajectories of 1g (blue), 4g (yellow), 7g (orange), 10g (red), and 13g (purple) flies at 24 hours post-hypergravity exposure. n=30 (1g); n=29 (4g); n=29 (7g); n=30 (10g); n=28 (13g).

Journal of Experimental Biology • Supplementary information

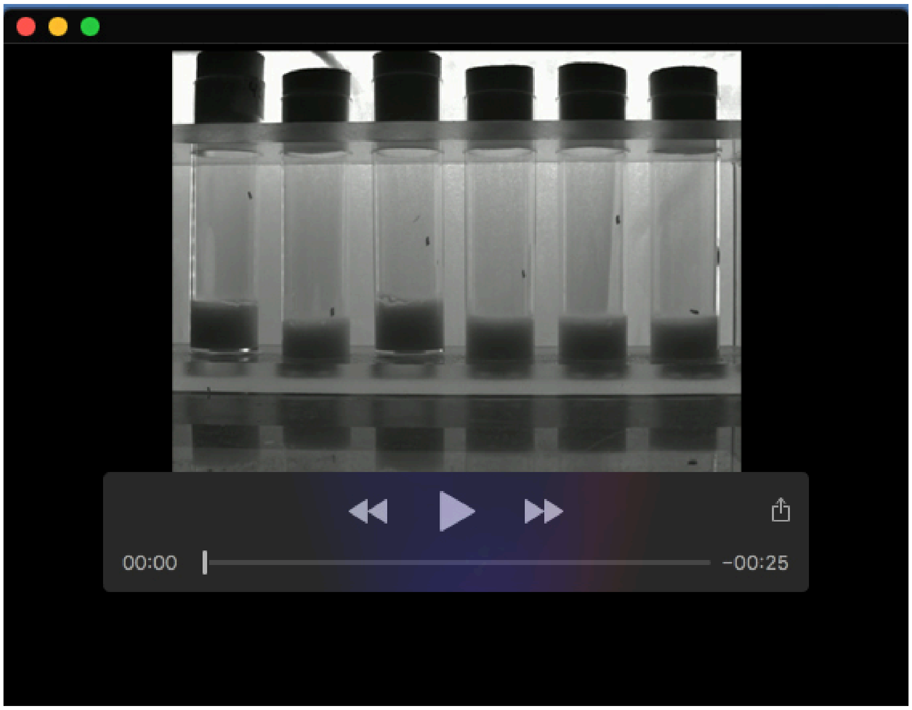

Movie 1. Startle-induced negative geotaxis (SING) assay for control flies (1g) for 4g. 6/6 flies climbed 5 cm within 15 seconds.

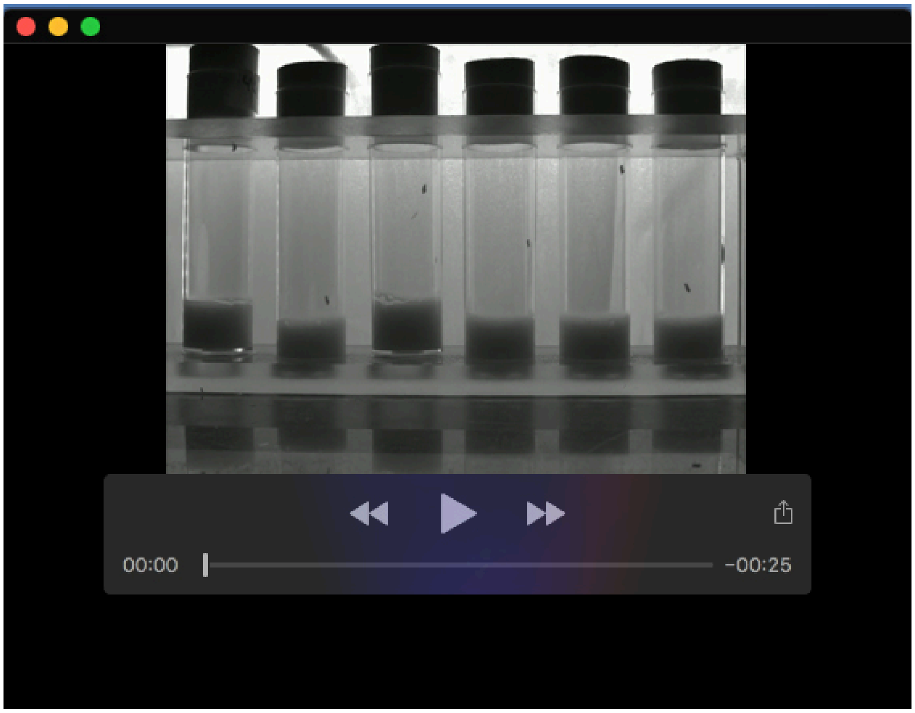

Movie 2. SING assay for 4g flies. 4/6 flies climbed 5 cm within 15 seconds.

Journal of Experimental Biology • Supplementary information

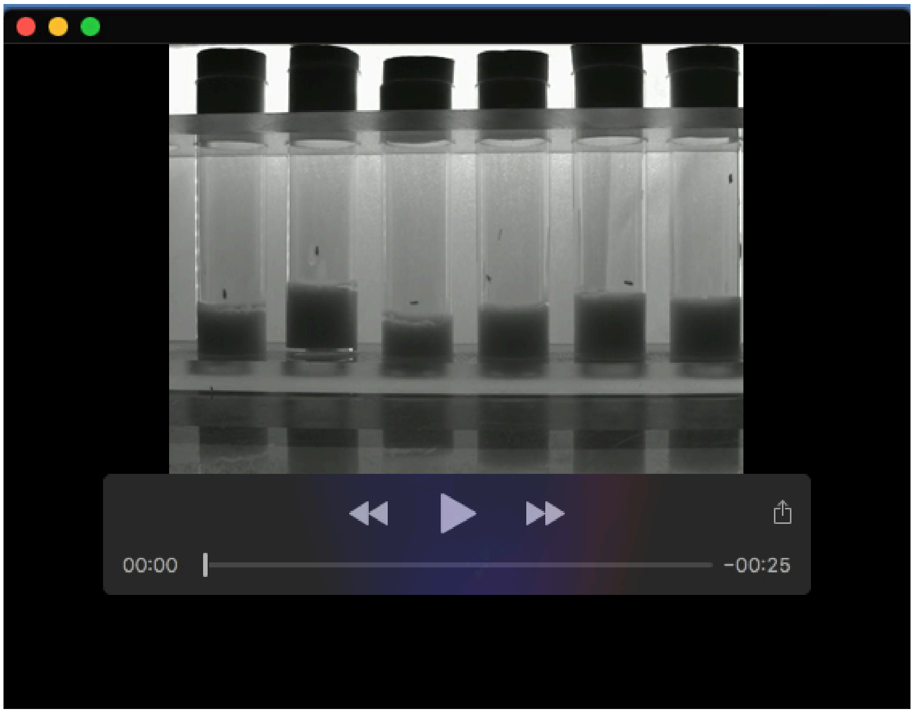

Movie 3. SING assay for control flies (1g) for 13g assay. All 6/6 flies climbed 5 cm within 15 seconds.

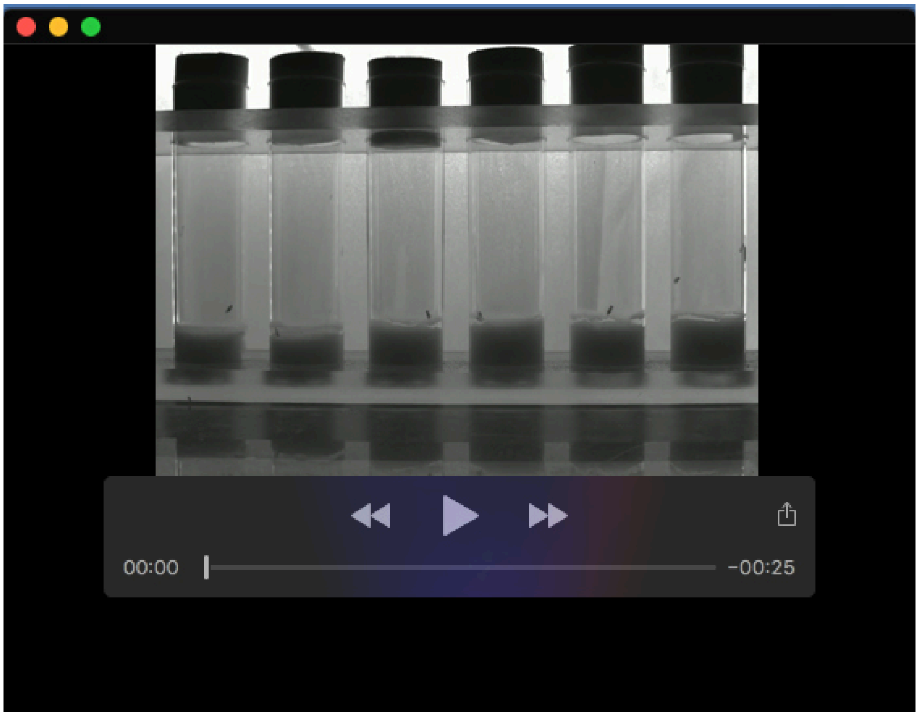

Movie 4. SING assay of 13g flies. 4/6 flies climbed 5 cm within 15 seconds.

Journal of Experimental Biology • Supplementary information

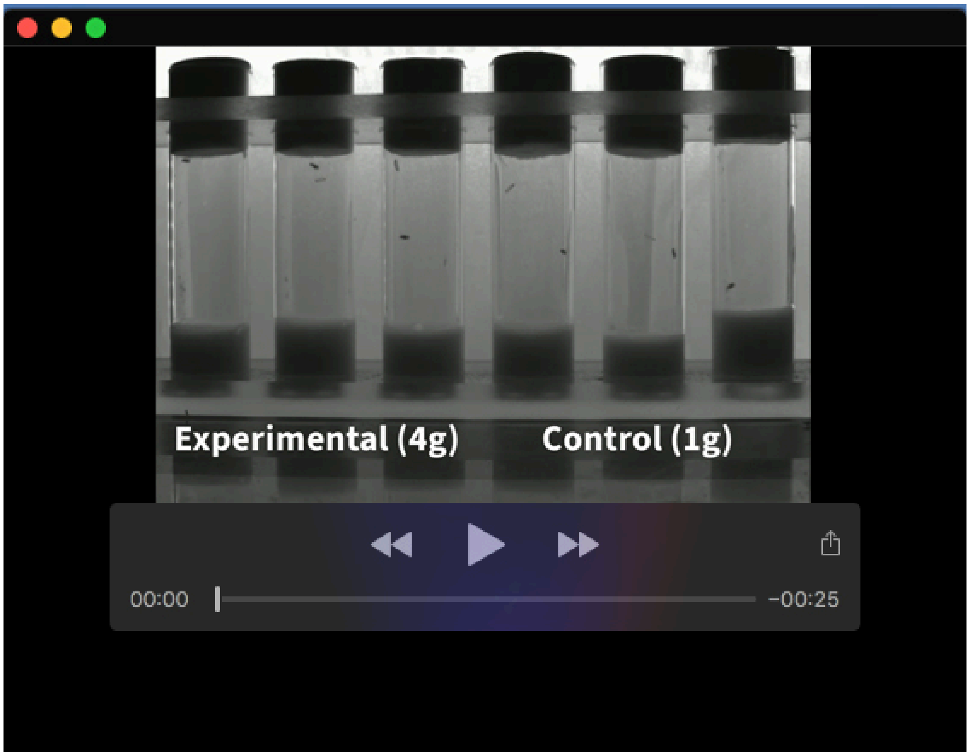

Movie 5. Negative geotaxis assay without startle for 4g flies. Flies were recorded for 5 minutes post-hypergravity exposure. The video is played back at 10× speed.

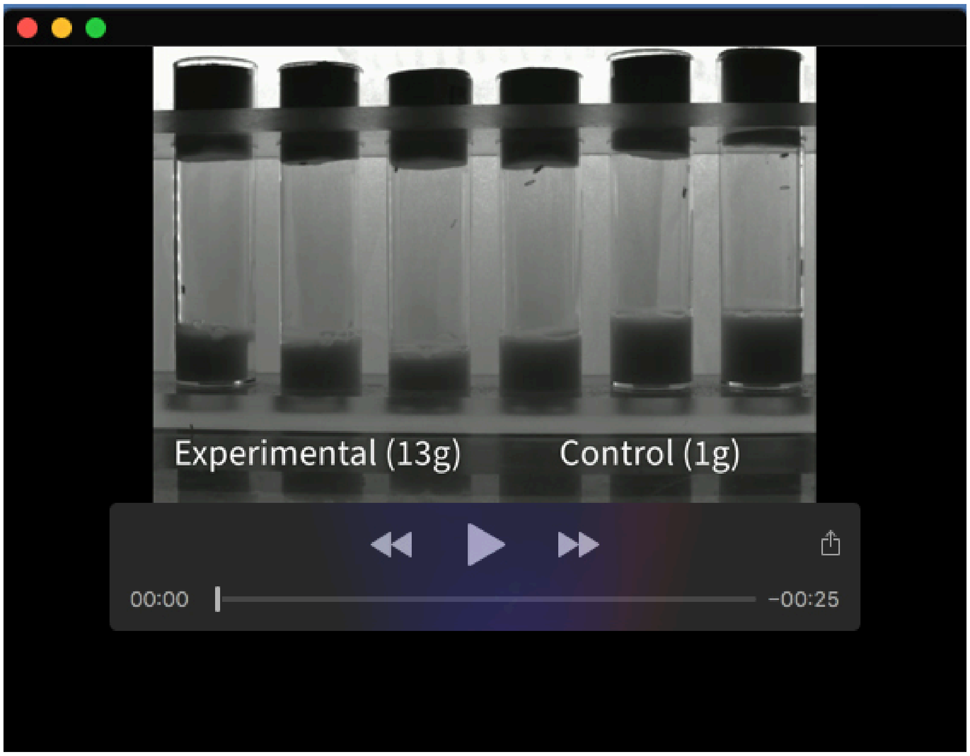

Movie 6. Negative geotaxis assay without startle for 13g flies. Flies were recorded for 5 minutes post-hypergravity exposure. The video is played back at 10× speed.
